# Characterization of mutations in the PAS domain of the EvgS sensor kinase selected by laboratory evolution for acid resistance in *Escherichia coli*

**DOI:** 10.1111/mmi.12704

**Published:** 2014-07-24

**Authors:** Matthew D Johnson, James Bell, Kim Clarke, Rachel Chandler, Prachi Pathak, Yandong Xia, Robert L Marshall, George M Weinstock, Nicholas J Loman, Peter J Winn, Peter A Lund

**Affiliations:** 1 Institute of Microbiology and Infection, School of Biosciences, University of BirminghamBirmingham, B15 2TT, UK; 2 College of Medical and Dental Sciences, University of BirminghamBirmingham, B15 2TT, UK; 3 Drug Delivery, Disposition & Dynamics, Monash Institute of Pharmaceutical Sciences381 Royal Parade, Parkville, 3062, Vic., Australia; 4 Institute of Integrative Biology, University of LiverpoolCrown Street, Liverpool, L69 7ZB, UK; 5 State Key Laboratory of Microbial Technology, Shandong University27 Shanda Southern Road, Jinan, China; 6 The Genome Institute, Washington University School of MedicineSt. Louis, MO, 63108, USA; 7 The Jackson Laboratory for Genomic MedicineFarmington, CT, 06030, USA

## Abstract

Laboratory-based evolution and whole-genome sequencing can link genotype and phenotype. We used evolution of acid resistance in exponential phase *Escherichia coli* to study resistance to a lethal stress. Iterative selection at pH 2.5 generated five populations that were resistant to low pH in early exponential phase. Genome sequencing revealed multiple mutations, but the only gene mutated in all strains was *evgS*, part of a two-component system that has already been implicated in acid resistance. All these mutations were in the cytoplasmic PAS domain of EvgS, and were shown to be solely responsible for the resistant phenotype, causing strong upregulation at neutral pH of genes normally induced by low pH. Resistance to pH 2.5 in these strains did not require the transporter GadC, or the sigma factor RpoS. We found that EvgS-dependent constitutive acid resistance to pH 2.5 was retained in the absence of the regulators GadE or YdeO, but was lost if the oxidoreductase YdeP was also absent. A deletion in the periplasmic domain of EvgS abolished the response to low pH, but not the activity of the constitutive mutants. On the basis of these results we propose a model for how EvgS may become activated by low pH.

## Introduction

The coupling of laboratory-based evolution by iterative selection with whole-genome sequencing is a powerful tool for dissecting aspects of cellular behaviour and the relationship between genotype and phenotype (recently reviewed in Brockhurst *et al*., 2011; Conrad *et al*., 2011; Dragosits and Mattanovich, 2013). Typical uses of this approach have been to identify genes important in giving *Escherichia coli* and *Saccharomyces cerevisiae* strains a competitive growth advantage under different defined conditions (e.g. Herring *et al*., 2006; Gresham *et al*., 2008; Barrick *et al*., 2009; Blaby *et al*., 2012), and to track changes in antibiotic resistance in pathogenic bacteria in the laboratory (Friedman *et al*., 2006; Wong *et al*., 2012) and in patients (Mwangi *et al*., 2007; Howden *et al*., 2011; Lieberman *et al*., 2011). In addition to the ability of such studies to yield insights into evolutionary processes, they have the further advantage that, as they rely on spontaneous mutation events, they require no presuppositions about which genes may be important for the processes under study. Thus this approach has great utility as an experimental tool.

The ability of organisms to respond to and survive different stresses often results from co-ordinated changes in large regulatory networks of genes. Acid stress in *E. coli* provides an example of this. *E. coli* is well adapted to survive low pH (which is likely to be related to its natural environment where en route to the gut it encounters stomach acid, bile acids, and short-chain fatty acids), and even laboratory strains of *E. coli* can survive several hours exposure to pH 2.5 if they are grown to stationary phase or if they are first exposed to a mild inducing acid shock of around pH 5.5 (Foster, 2004). At least four different systems are involved in regulating resistance to low pH, and their regulation involves the action of two-component systems, regulatory proteins, small RNAs, and both post-transcriptional and post-translational events. For example, the best understood acid resistance system in *E. coli*, known as AR2 or the GAD system, involves members of at least three two and three-component regulator systems (EvgAS, RcsB, and PhoPQ; Masuda and Church, 2002; Castanié-Cornet *et al*., 2007; Eguchi *et al*., 2011), a further four regulatory proteins, which are not completely specific to the acid resistance networks (YdeO, GadE, GadX, and GadW; Shin *et al*., 2001; Ma *et al*., 2002; 2003[Bibr b49],[Bibr b50]; Masuda and Church, 2002; Tramonti *et al*., 2002), a small regulatory RNA (gadY; Opdyke *et al*., 2004), and a protease (Lon; Heuveling *et al*., 2008). Much has been learnt about the regulatory circuitry underlying acid resistance, but to connect the molecular architecture of this to the whole organism's response to low pH, and to the process of evolutionary adaptation, whole organism studies using a combination of post-genomic and systems biology tools are required. Indeed, such studies have already yielded new insights into acid resistance mechanisms (Burton *et al*., 2010; King *et al*., 2010; Stincone *et al*., 2011).

Here, we have studied acid resistance in *E. coli* by using an evolutionary approach combined with whole-genome sequencing. Rather than adapting strains to a gradual decrease in pH, we used cycles of growth following exposure to near bactericidal levels of acid. We show that, although a wide range of regulators control the response to low pH, evolution of a constitutive acid resistance phenotype during exponential phase growth was always associated with mutations in the PAS domain of the EvgS membrane sensor-kinase protein. These mutations lead to wholesale activation of many genes for acid resistance. We further demonstrate that low pH is detected by the periplasmic domain of EvgS, that there is a surprising level of redundancy in the activated pathway, and propose a mechanism for how structural changes in an EvgS dimer may lead to its activation.

## Results

### Iterative selection at pH 2.5 during exponential phase rapidly leads to acid resistance

**Figure 1:**
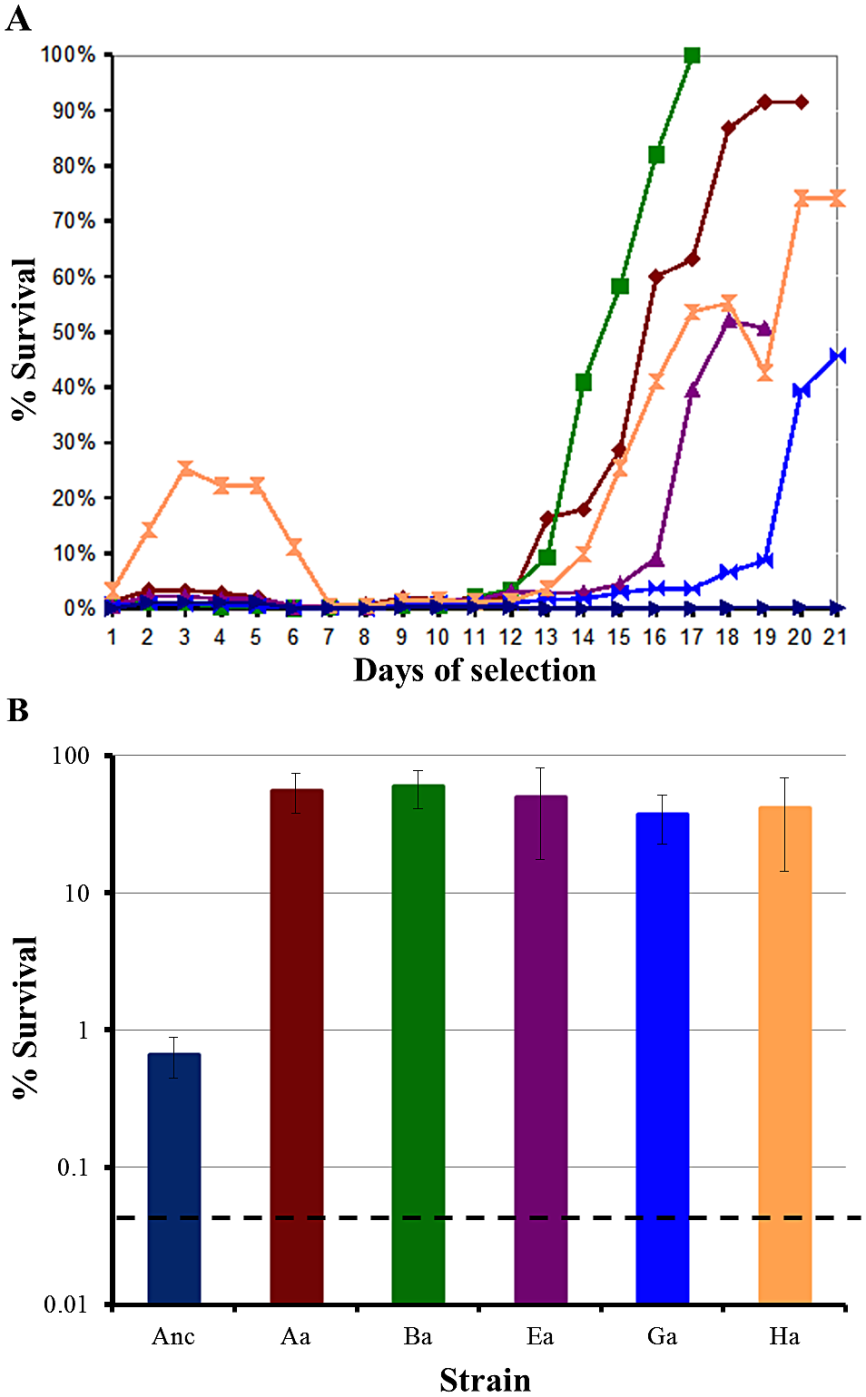
Evolution of the acid-resistant phenotype.A. Five independent cultures of *E. coli* K-12 MG1655 were iteratively exposed to a pH of 2.5 in exponential phase over 20 days, and their survival was measured. The cell lines were labelled A (red), B (green), E (purple), G (light blue), and H (orange). A control cell line, which was not exposed to pH 2.5, was also assayed (dark blue). Percentage survival was measured as an average over four consecutive days (thus, the values for day 1 are the average of the values for days 1–4). Experiments on each line ended on different days.B. Individual colonies were isolated from each culture at the end of the experiment, and tested for acid resistance. Results for colonies that showed high levels of resistance (one from each independent culture) are shown compared to the ancestral starting strain. The clonal isolates from cultures A–H were named Aa–Ha respectively. The error bars are the standard deviations of three independent biological replicates.

**Figure 2:**
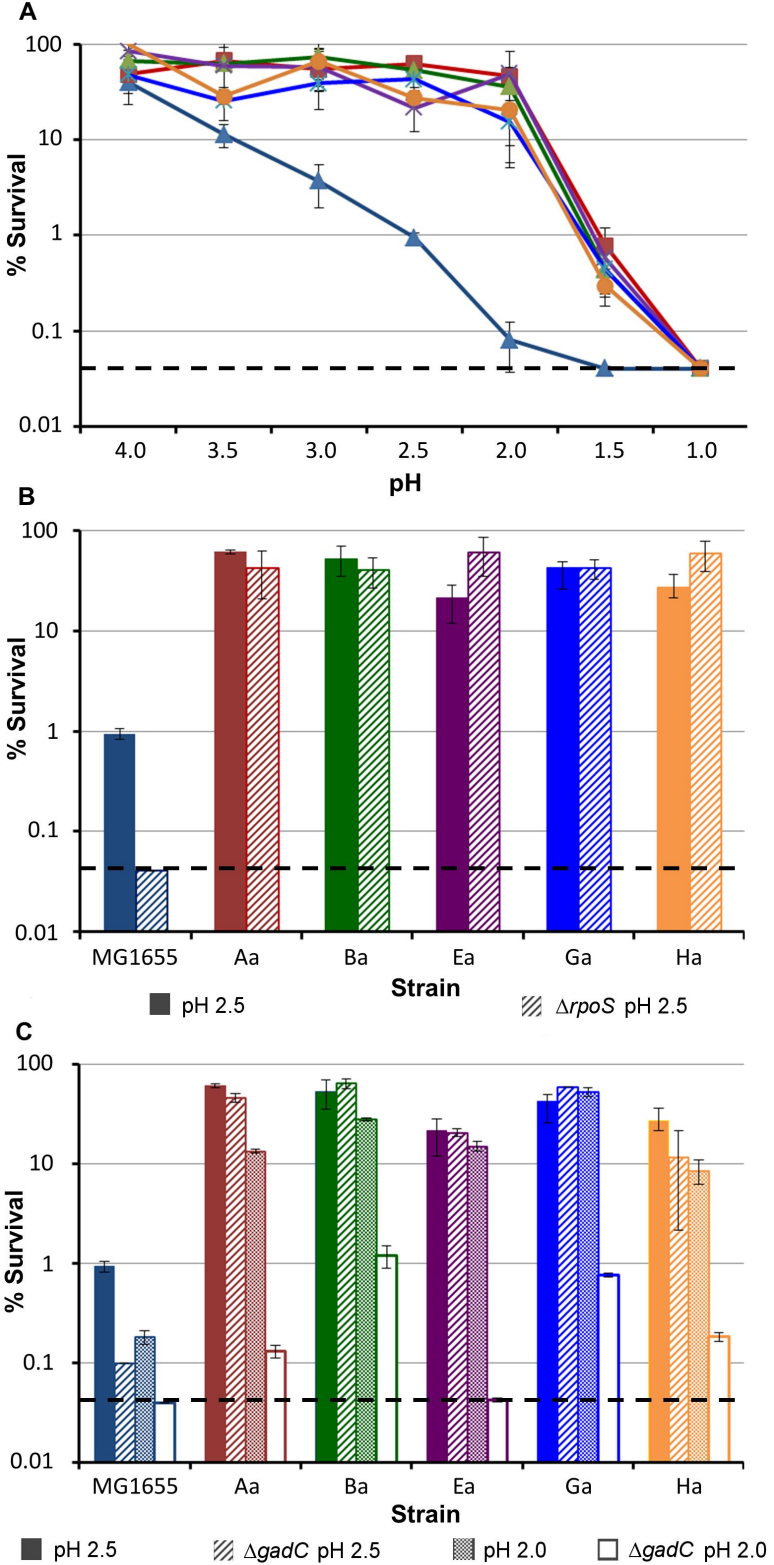
Comparison of evolved strains in different pH conditions and genetic backgrounds.A. Survival of each clonal isolate (Aa–Ha), and the MG1655 ancestor, after 2 h at different levels of acidity. Strains are shown with the same colour scheme as Fig. 1.B. Comparison of acid resistance at pH 2.5 of the ancestor and evolved strains in wild-type (solid bars) and Δ*rpoS* (diagonal hatched bars) backgrounds.C. Comparison of acid resistance of the ancestor and evolved strains in wild-type background at pH 2.5 (solid bars) or pH 2 (crosshatched bars), and Δ*gadC* background at pH 2.5 (diagonal hatched bars), or pH 2 (untextured bars). Percentage survival was calculated by dividing the number of colonies after 2 h in acid by the number of colonies at time zero at pH 7. The black dotted line represents the limit of detection at 0.04%. Error bars show the standard deviations of three independent biological replicates.

Measurement of the level of acid resistance of the cultures that were iteratively exposed to acid showed that after an initial lag when the cultures were uniformly sensitive to low pH, resistance began to appear, generally around 12 days after selection had begun (Fig. 1A). An early transient acid resistance phenotype seen in culture H was not further investigated. The control culture showed no development of acid resistance. The levels of resistance reached in the different cultures varied from approximately 100% to 40%. Several single colonies from each culture were isolated and screened, and one from each culture that showed high resistance was used for all future experiments. These were labelled MG1655-Aa, Ba, Ea, Ga and Ha. Levels of survival of these strains after 2 h at pH 2.5 are shown in Fig. 1B, and varied between 59% and 37%, compared to the starting strain MG1655 (which we refer to as the ancestor strain) which showed approximately 1% survival under identical conditions. This high level of resistance was preserved at all phases of growth, and was consistently higher than that shown in the ancestor even in stationary phase (Fig. S1). The evolved strains showed significantly higher exponential phase acid resistance than the ancestor over a range of pH values, starting at approximately 3.5 (Fig. 2A). All the evolved strains showed some survival after 2 h even at pH 1.5, whereas no survival of the ancestor was seen at this extremely low pH.

Slowing of growth rate may cause an increase in RpoS levels, which could lead to increased acid resistance (Ihssen and Egli, 2004), so we compared the growth rates of all strains in the medium used to assess acid resistance. In exponential phase, the doubling times for the evolved strains were between 40 and 43 min, compared to 35 min for the wild-type ancestor. No increase in acid resistance was seen when the growth rate of the ancestor was decreased to below that of the evolved strains by using a less rich medium (not shown). Deletion of *rpoS* from the evolved strains also did not lead to any significant change in their levels of acid resistance in exponential phase, although the resistance of the ancestor declined from around 1% survival after 2 h at pH 2.5 to less than 0.01%, showing that *rpoS* plays some role in acid resistance in exponential phase MG1655 (Fig. 2B), but not in the mutants.

Conditions in the selection experiment were deliberately chosen so as not to select specifically for mutations in any of the known acid resistance systems of *E. coli* (AR1 to AR4; Foster, 2004). As the major determinant of acid resistance in exponential phase is the decarboxylation of glutamate, we examined the effect of deleting *gadC*, which encodes the essential glutamate-GABA antiporter, on the phenotype of the acid-resistant strains. As expected, deletion of this gene in the MG1655 ancestor led to a significant decrease in acid resistance, at both pH 2.5 and pH 2. At pH 2.5 this deletion had no effect on the resistance of the evolved strains, but absence of *gadC* gave significantly reduced resistance at pH 2, showing the GAD system to be required for resistance of the evolved strains at this lower pH, but not at the pH at which they were selected (Fig. 2C). Growth on other amino acids which can contribute to acid resistance (arginine and lysine) had no effect on resistance of the evolved strains (not shown).

**Figure 3:**
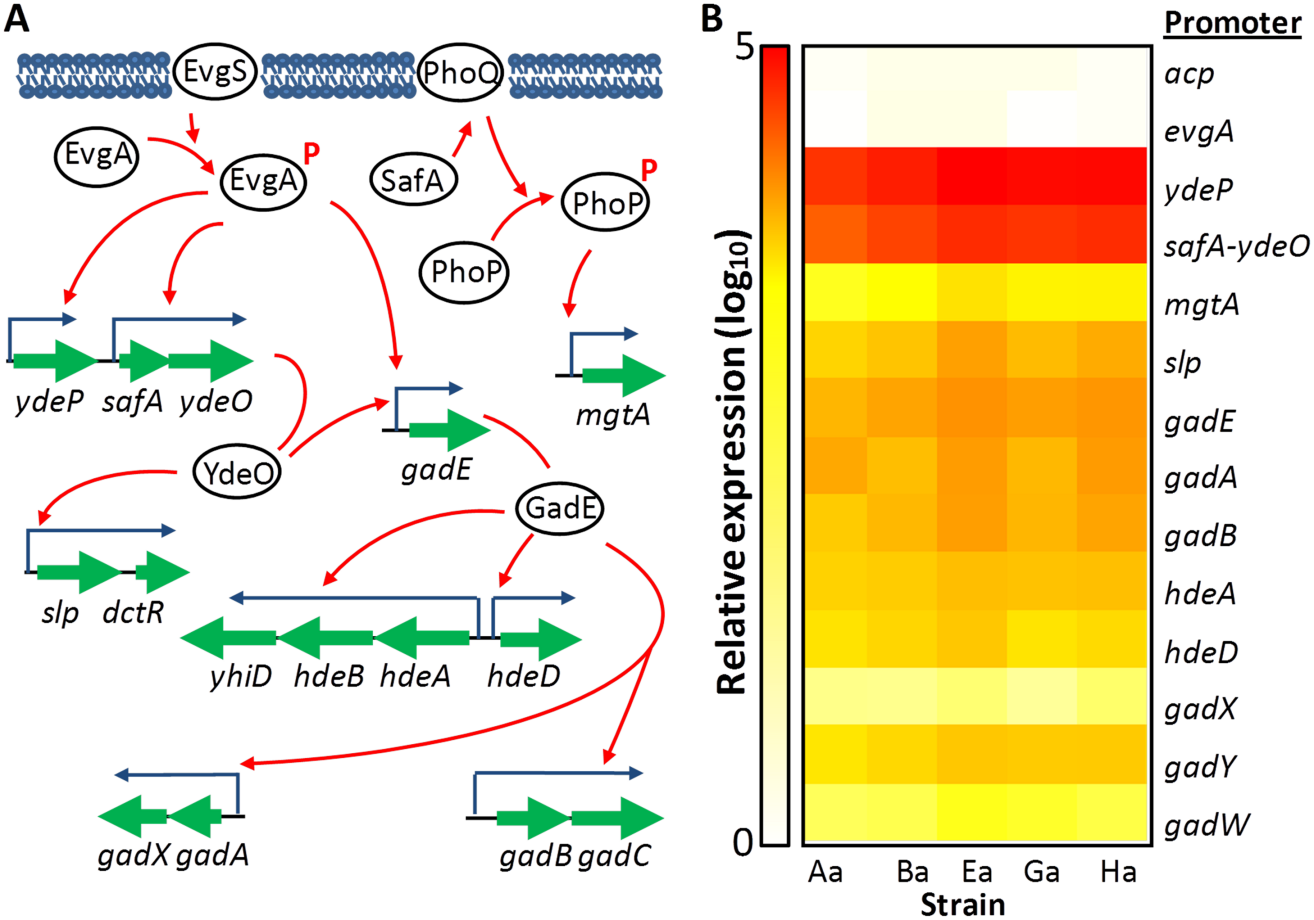
Activities of promoters in the AR2 system in the acid-resistant strains.A. Simplified diagram of the regulatory relationships between the different components in the AR2 system that were investigated in this study. Red arrows indicate activation. Known repression circuits are omitted for clarity.B. Levels of expression for all the named promoters in all five of the evolved acid-resistant strains, as measured by a luciferase reporter gene. Levels are expressed relative to the activities of the same promoters measured in the ancestor MG1655 strain under identical conditions. The *acpP* promoter is a control promoter which is not part of the AR2 system.

**Figure 4:**
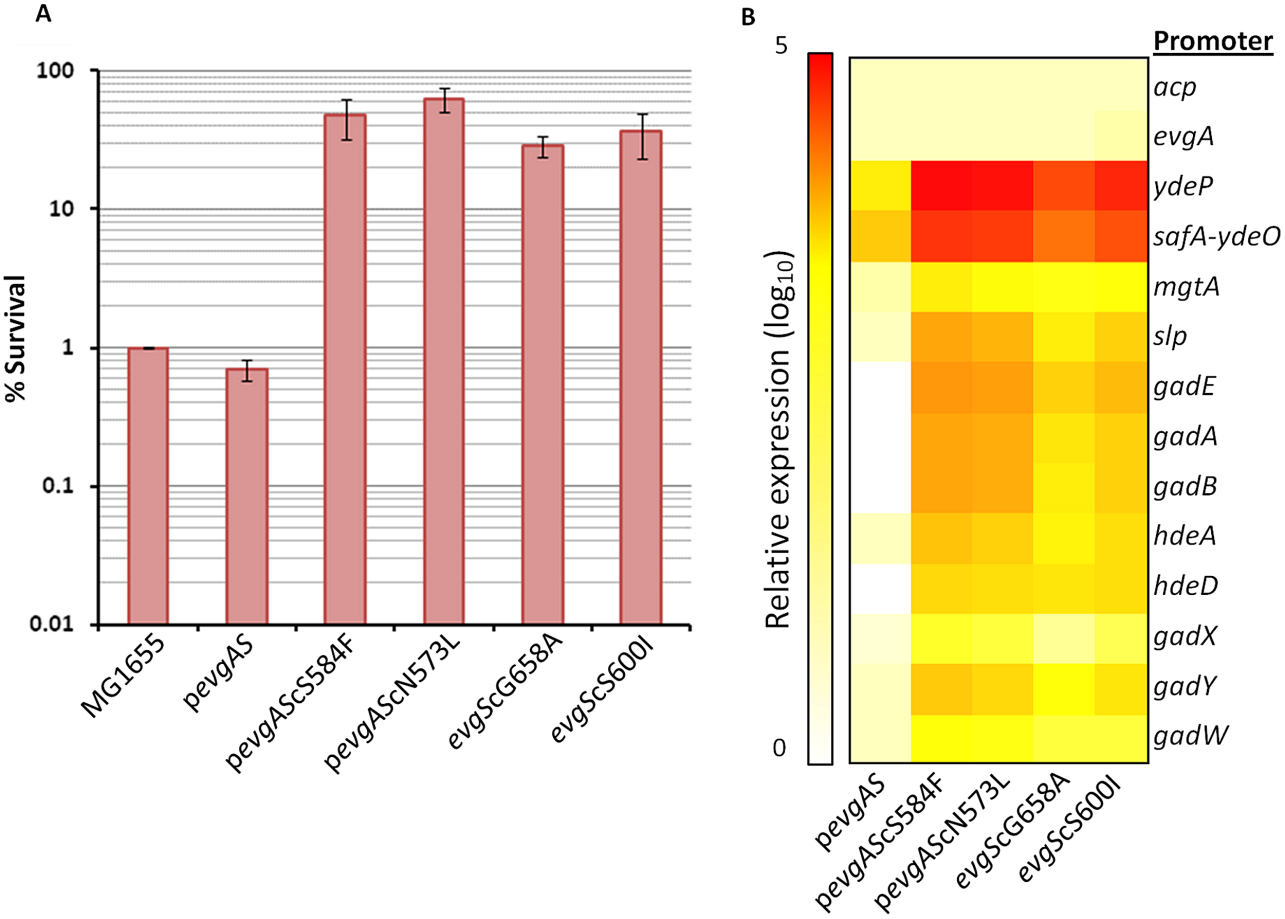
Levels of acid resistance and promoter activities associated with the *evgS* mutations.A. survival at pH 2.5 for 2 h was determined for the ancestral MG1655 strain, and for the same strain containing plasmids carrying wild-type or mutant *evgS*, or with mutated *evgS* genes inserted into the chromosome.B. levels of expression for all the named promoters in the presence of the wild-type or mutated evgS genes, either on plasmids or on the chromosome.

Because of the indication that the AR2 system played a role in the evolved phenotype, we examined the promoter activities of 13 different genes involved in this system, using luciferase reporters (described in Burton *et al*., 2010). All the promoters showed significant upregulation in all the evolved strains at pH 7 compared to the wild-type strain (Fig. 3; raw data for both Fig. 3 and Fig. 4 are shown in Table S3), apart from the promoter of the *evgAS* operon, which is known not to be regulated by acid, and of *acpP*, a control gene which is well expressed and which we had previously shown is not acid regulated (N. Burton, unpublished). This expression profile is consistent with one that is seen in cells which have been exposed to mild acid, which is known to induce high tolerance to a subsequent severe acid treatment (Goodson and Rowbury, 1989; Tucker *et al*., 2002).

### The acid resistance phenotype is caused by mutations in *evgS*

We determined the complete genome sequence of four of the acid-resistant strains (Aa, Ba, Ea and Ga) in parallel to that of the MG1655 ancestor. The results (Table S2) showed that although each strain contained distinct mutations in 3 to 10 different genes, each contained a unique mutation in the *evgS* gene. The *evgAS* operon in the fifth strain was also sequenced, and was found to contain the same mutation as in Ga; other sequences in Ha showed that Ha and Ga were independent and not descended from the same mutational event (data not shown). EvgS is part of the EvgAS two-component system that has been implicated in the response to low pH in *E. coli* (Masuda and Church, 2002; 2003; Ma *et al*., 2004; Itou *et al*., 2009), so this suggested that these strains might have become resistant by constitutive activation of the *evgAS* regulon even in the absence of a low external pH. This result was also consistent with the data shown in Fig. 3B, which shows elevated expression of the genes of the EvgAS regulon in the absence of acid. We therefore refer to these mutations generically as *evgS^c^* mutations.

To test whether each of the mutations in the *evgS* gene was sufficient for the resistant phenotype, all four mutated *evgS* genes were reintroduced into the MG1655 ancestor, either by cloning on the low-copy-number plasmid pZC320 (Shi and Biek, 1995) for EvgS-S584F and EvgS-N573K, or by replacement of the wild-type allele by gene doctoring (Lee *et al*., 2009) for EvgS-G658A and EvgS-S600I. In all cases, the strains carrying the mutated *evgS* alleles had acid-resistant phenotypes that were indistinguishable from those of the evolved strains (compare Figs 1B and 4A). The levels of the same acid-regulated and control promoters were also consistent with those found in the original evolved strains (compare Figs 3B and 4B). The mutated *evgS* genes are thus largely if not solely responsible for the acid-resistant phenotype. The phenotype is predicted to be completely EvgA dependent, and deletion of *evgA* indeed led to loss of the acid resistance in the strain expressing EvgS-G658A (Fig. S2). When EvgS is activated, it becomes autophosphorylated at his721. The constitutive activity of the evgS^c^ mutants should thus be abolished if this residue is mutated to a glutamine. To test this, we measured the activity of a chromosomally encoded p*ydeP–lacZ* fusion in the presence of plasmid-encoded variants of EvgS under the control of a pBAD promoter, in a strain where the wild-type *evgS* gene had been deleted. (EvgS expressed from these plasmids was his-tagged for other experiments not reported here; this has no effect on its ability to activate *ydeP* expression. The strain and plasmid for this experiment were a generous gift of Prof. Ryutaro Utsumi and Dr Yoko Eguchi, Kinki University, Japan). As predicted, mutation of *evgS* his721 to gln abolished the acid inducibility of the *ydeP* promoter, and also abolished the constitutive activity of evgS^c^ S584F and S600I (Fig. S3). The growth rates of strains carrying the double mutants *evgS*^c^ H721Q were also restored to normal (not shown).

To see whether other properties of the strains were altered by the acid resistance phenotype, we carried out a complete phenotypic analysis and comparison of the evolved strain carrying the *evgS*^c^ G658A mutation, the doctored strain carrying just this mutation, and the wild-type ancestor strain, using the phenotype microarray (Biolog) system (Bochner *et al*., 2001; Bochner, 2009), which tests over 2000 different conditions including different C, N, P and S sources, and a wide range of stress conditions, for their effects on bacterial growth. No significant differences between the strains were detected by this method (data not shown). We also looked to see whether the strains showed resistance to high temperature. All the evolved strains, and the strain containing only the *evgS^c^* G658A mutation, showed a small but reproducible improvement in their ability to survive incubation at 51°C (not shown) compared to the ancestor. This is interesting in light of the demonstration that overexpression of *evgA* leads to enhanced temperature resistance above 50°C (Christ and Chin, 2008).

To investigate the global effects of constitutive activation of *evgS* on gene expression, we analysed mRNA extracted from the strain carrying one of the *evgS* mutations (S600I) by microarray. The results are summarized in Table S4. One hundred and eighty-eight genes showed a log_2_-fold induction of more than 2 with a FDR of < 1%. Forty-two of these genes were also seen in the array analysis of a previously reported constitutive mutant of *evgS* (Eguchi *et al*., 2003), and the relative levels of expression of these genes correlated well between the two data sets, but there were also significant differences between the two lists, possibly due to the different natures of the mutations in *evgS,* the different growth conditions, or different strain backgrounds in the two studies.

Forty-nine of the genes in our list had a log_2_-fold induction greater than 4. Among the most highly expressed genes are most of the major genes of the AR2 system, as expected. Additional genes which have also been shown previously to be *evgA* regulated were also seen including the drug transporter *emrKY*, and two genes involved in detoxification of oxalate: *frc* (or *yfdW*) (which showed the highest fold increase) and *oxc* (or *yfdU*). Many more genes show activation of expression in this mutant than those of the AR2 system alone. Many of the most strongly upregulated genes have roles in nitrogen metabolism, and also in other cellular processes including iron metabolism and membrane transport. We searched the upstream regions of the promoters of all the genes whose log_2_-fold expression increased by more than fourfold for evidence of an EvgA binding site, which is found in all of the genes which are known to be directly regulated by EvgA, but no sites were found except in those genes which have already been shown to be EvgA-regulated, so we conclude that these genes are likely to be overexpressed in the presence of the *evgS^c^* mutation due to indirect effects. The fact that many genes are changed in expression level, but the phenotype array shows no significant differences in phenotypes tested, probably reflects the fact that gene expression data are still often a rather poor predictor of phenotype (Xu *et al*., 2009).

### The constitutive acid resistance phenotype reveals functional redundancy in the AR2 system

EvgS acts by phosphorylation of EvgA, which in turn regulates the genes of the AR2 system, including the glutamate decarboxylases *gadA* and *gadB*, and the gene for the glutamate/GABA antiporter *gadC* (Fig. 3A). EvgA activates the gad system directly by activating expression of the central regulator GadE, and indirectly in two ways: one via activation of YdeO which activates GadE, and the other via activation of a PhoP/PhoQ-dependent pathway that activates GadE via RpoS (Eguchi *et al*., 2011). Our array data showed clearly that the constitutive mutation in EvgS activated the YdeO–GadE pathway, which provides a potential explanation of the acid resistance phenotype of these mutants. The array data showed many other changes taking place in the levels of expression of genes which are not known to be related to the AR2 system. To determine the importance of the AR2 system in the observed acid resistance, we therefore compared the effects of a number of different gene deletions in this system both on the *evgS*^c^-induced acid-resistance phenotypes, and on inducible acid resistance in cells with wt *evgS*.

Deletion of the *rpoS* and *phoP* genes led to a significant drop in both non-induced and pH 5.5-induced acid resistance in the MG1655 ancestor, as expected. The presence of a plasmid carrying the wild-type *evgAS* operon had no effect on the non-induced acid resistance of this strain, but in the presence of the same plasmid carrying an *evgS*^c^ mutation (N573L), non-induced acid resistance to pH 2.5 or pH 2.0 was shown to be very high and not significantly different from that caused by this allele in the wild-type background (Table 1). Thus although a small effect is evident, the PhoPQ pathway does not play a substantial role in resistance mediated by constitutively activated EvgS under the experimental conditions that we used. We hypothesized that loss of *gadE,* the central regulator of the AR2 pathway*,* would have a more severe effect, but in fact loss of *gadE* had no significant effect at pH 2.5, although it was needed for the non-induced and induced phenotypes in the wild-type strain. We confirmed that deletion of *gadE* led to loss of EvgS^c^-dependent expression of the *gadB* and *hdeA* promoters, to ensure that activation of the *gadE*-regulated genes was not taking place via a different route in the *evgS*^c^ background (data not shown). At pH 2.0 however the EvgS^c^-mediated phenotype was completely dependent on *gadE* (Table 1). YdeO regulates several genes with a role in acid resistance that are not directly regulated by GadE but deletion of *ydeO* also had no effect at pH 2.5, and only a modest effect at pH 2.0. A double deletion of both *ydeO* and *gadE* had essentially the same effect on EvgS^c^-mediated acid resistance as a *gadE* deletion, as predicted from the results above (Table 1).

**Table 1:** Effects of different genetic backgrounds on acid resistance in MG1655

Mutations/plasmids	Condition	% survival, pH 2.5	% survival, pH 2.1
MG1655 wild type	U	0.6	0.6
MG1655 wild type	I	16	Nd
MG1655/pevgAS^c^N573L	U	55	82
Δ*rpoS* Δ*phoP*	U	< 0.04	Nd
Δ*rpoS* Δ*phoP*	I	5	Nd
Δ*rpoS* Δ*phoP*/pevgAS	U	< 0.04	Nd
Δ*rpoS* Δ*phoP/*pevgAScN573L	U	29	Nd
Δ*gadE*	U	< 0.04	Nd
Δ*gadE*	I	0.08	Nd
Δ*gadE/pevgAS*	U	< 0.04	Nd
Δ*gadE/*pevgAS^c^N573L	U	40	< 0.04
Δ*ydeO*	U	0.2	Nd
Δ*ydeO*	I	0.8	Nd
Δ*ydeO/pevgAS*	U	0.2	Nd
Δ*ydeO/*pevgAS^c^N573L	U	37	11
Δ*gadE* Δ*ydeO*	U	< 0.04	Nd
Δ*gadE* Δ*ydeO*	I	0.06	Nd
Δ*gadE* Δ*ydeO/pevgAS*	U	< 0.04	Nd
Δ*gadE* Δ*ydeO/*pevgAS^c^N573L	U	26	Nd
Δ*ydeP*	U	0.4	Nd
Δ*ydeP*	I	7	Nd
Δ*ydeP/pevgAS*	U	0.5	Nd
Δ*ydeP/*pevgAS^c^N573L	U	55	75
Δ*gadE* Δ Δ*ydeP*	U	< 0.04	Nd
Δ*gadE* Δ*ydeO* Δ*ydeP*	I	< 0.04	Nd
Δ*gadE* Δ*ydeO* Δ*ydeP/pevgAS*	U	< 0.04	Nd
Δ*gadE* Δ*ydeO* Δ*ydeP/*pevgAS^c^N573L	U	< 0.04	Nd

U, uninduced; I, induced by incubation at pH 5.5; Nd, not determined. % survival is after 2 h exposure, and is expressed relative to the colony count at t = 0.

The only other EvgS-regulated component of AR2 known to have a role in acid resistance is *ydeP* which is adjacent to *safA* and *ydeO* (Masuda and Church, 2003). Deletion of the *ydeP* gene reduced the induced resistance of the MG1655 ancestor, but had no effect on the resistance of the strain carrying *evgS*^c^N573 (Table 1). This result eliminated the possibility that any one component of the EvgS-regulated AR2 system was necessary and sufficient for resistance at pH 2.5 in the *evgS*^c^ mutants. This left two possibilities: either some other gene or pathway is needed for acid resistance, or different components of the AR2 system can substitute for each other to give an acid-resistant phenotype at pH 2.5. To test this latter hypothesis, we constructed a triple deletion mutant lacking *ydeP*, *gadE*, and *ydeO*. This mutant now failed to show any detectable resistance even in the presence of evgS^c^N573L at pH 2.5. Thus the acid resistance phenotype caused by the *evgS*^c^N573 mutation can be mediated either by overexpression of YdeP alone, or by overexpression of one or more YdeO and/or GadE-regulated genes, revealing an unexpected level of redundancy in the AR2 system.

### Additional mutations that activate EvgS are all found in the PAS domain

**Figure 5:**
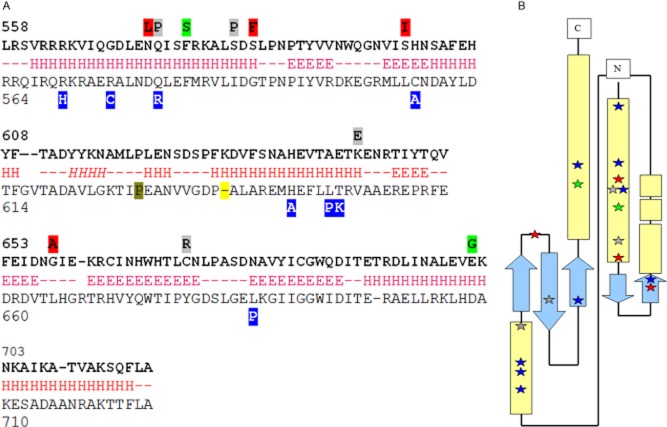
Position of mutants that activate EvgS and BvgS.A. The putative PAS domains of EvgS (top line) and BvgS (bottom line) were aligned, and positions of known activating mutations are shown above the line (EvgS) or below it (BvgS). Those highlighted in red were found in the evolved populations; those highlighted in grey were found by direct selection on evgS; and those highlighted in green have been described previously (Kato *et al*., 2000). BvgS mutations are shown in blue. The secondary structure of the PAS domain of EvgS was predicted using psipred, PHYRE2, and jpred2, and these predictions are shown below the EvgS sequence. Predictions were close between all three methods apart from the helix shown in italics, which was predicted by psipred and jpred2 but not PHYRE2.B. Diagram of potential structure of the PAS domain of EvgS, showing the 5-stranded anti-parallel beta-sheet with mutations marked using the same colour scheme as in A. Regions predicted to form beta-strands are light blue arrows; alpha helices are light yellow boxes.

Prediction of domain structure for EvgS and its homologue BvgS using the SMART online server (Schultz *et al*., 2000; see Fig. S4) reveals several domains in common. BvgS, but not EvgS, is predicted to have a single PAS domain immediately downstream of the transmembrane region. However, structural predictions on the EvgS region corresponding to the BvgS PAS domain suggest that it also has the characteristics of a PAS fold, and we therefore refer to it here as the EvgS PAS domain. All the mutants found in this evolutionary approach mapped in the EvgS PAS domain. Two other mutations activating EvgS in the absence of acid have been reported previously (Kato *et al*., 2000), and these also map in the PAS domain (Fig. 5). BvgS is usually activated, but responds to negative modulators such as sulphate and nicotinate; mutations that perturb this response also map in the PAS domain (Miller *et al*., 1992; Goyard *et al*., 1994; Manetti *et al*., 1994; Dupré *et al*., 2013). We looked for more mutants in *evgS* to see whether this pattern would be consistent using a more direct system to select for such mutants. We constructed the plasmid p*ydeP-tet* which contains a tetracycline resistance gene under the control of the *ydeP* promoter, which is strongly upregulated from a very low level by activated EvgA (Burton *et al*., 2010). The presence of this plasmid in the same cell as either an empty vector or a plasmid carrying the wild-type *evgAS* operon did not cause tetracycline resistance, but if a plasmid carrying one of the constitutive *evgS* mutations was introduced into the cell, the cells became resistance to approximately 6 μg ml^−1^ tetracycline, which provides a useful selection for novel *evgS* mutants. We passaged the plasmid pBAD-evgS-his through the XL1-red mutator strain, introduced it into a strain carrying the pydeP-tet plasmid, and selected for growth on 6 μg ml^−1^ tetracycline. A further four independent point mutations were found using this approach, and all mapped in the PAS domain of *evgS* (see Fig. 5A). All caused acid resistance at pH 2.5 (data not shown), and activated a p*ydeP–lacZ* fusion at pH 7 (Fig. S5). Thus although constitutive mutations may still be found that map elsewhere in the protein, it is clearly the case that mutations in the PAS domain are the most likely to activate the protein.

To map the positions of these mutations in the PAS domain, we used different methods to predict the secondary and tertiary structure of this domain (summarized in Fig. 5A). Most of the mutations that activate EvgS are found in the helical cap at the N-terminal end of the domain; this is also true for the mutations that have been found to activate the homologue, BvgS (Fig. 5B), although not all activating mutations are found in this region. We produced a comparative model of the EvgS structure, guided by secondary structure prediction (summarized in Fig. 5A), the experimentally determined structure of a number of PAS domains, and a sequence alignment of these domains (Fig. S6). Although there is considerable sequence variation between the different PAS domains, the alternating hydrophobic/hydrophilic pattern common to beta strands along with the periodic pattern of an alpha helix and a few totally conserved residues, notably glycine in the loop between strand 1 and 2 of all these PAS domains, a conserved asparagine at the C-terminal end of the second strand, and a conserved D I/A/V T triplet at the C-terminal end of the fifth strand, allowed us to produce a sequence alignment with confidence in the alignment of the beta-strands. The sequence alignment of the alpha-helices is less certain, reflecting the marked variability in alpha helix position between the known 3D structures that we looked at.

**Figure 6:**
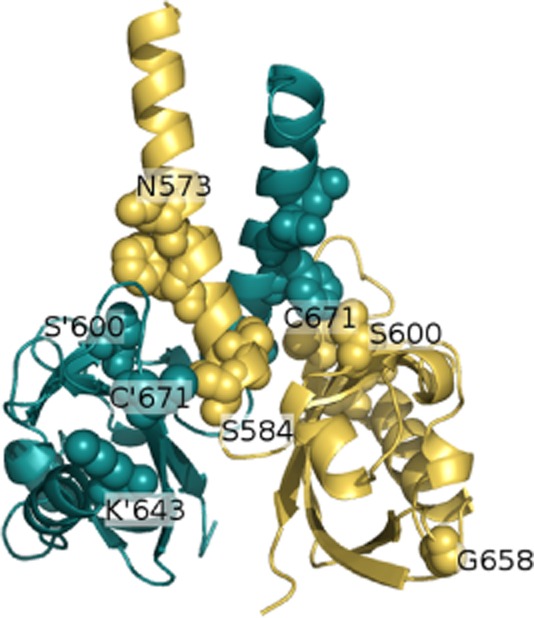
Comparative model of the EvgS PAS domain dimer. One chain is coloured as a blue-green ribbon, the other a gold ribbon, the side-chains of residues where mutation leads to constitutive acid resistance are shown as spheres. Residues mentioned in the text are labelled, except for G701 which is C-terminal to the model, which would place it at the bottom of the figure. A prime (′) next to an amino acid code indicates a residue on the blue-green chain, e.g. C′671 is on the blue chain at the foreground left of the figure, whereas C671 is on the gold chain, just behind S600. It is evident that the dimer is approximately symmetric around a C2 axis. The majority of the mutants lie at the interface between the N-terminal helix of one chain and the beta-sheet and loops of the other. See Fig. S7 and *Experimental procedures* for further details.

Analysis of the surface properties of a monomeric EvgS PAS domain model (Fig. S7 (b)) combined with PIER analysis were consistent with formation of a dimer similar to two crystal structures already solved (the PAS domain of the halobacterial transducer rhodopsin (HTR)like protein from *Haloarcula marismortui* (PDB ID 3BWL) and the PAS domain from the VicK protein of *Streptococcus mutans*, PDB ID 4I5S; Wang *et al*., 2013). These dimers consist of a coiled-coil formed by the N-terminal helices of the monomers, and then an interaction between the beta sheet of one monomer and the N-terminal helix of the other monomer (Fig. 6, Fig. S7). A model of the EvgS dimeric structure indicated that most of the mutations that activate EvgS are at the interface between the coiled-coil and the beta-alpha domain (Fig. 6), with four possible exceptions. These are N573, arguably at this interface but clearly on the periphery; K643, which is immediately adjacent to C671, which is the site of an activating mutation that contacts the interface, and G658A and G701E, which both lie well away from the interface. A simple structural explanation for these results is thus that the non-active structure contains two EvgS monomers, held together in an inactive conformation, and that activation of the EvgS protein alters the interaction between the two monomers and causes EvgS to become active as an autokinase. The mutations in the PAS domain may mimic this structural change by altering or dissociating the inactive dimer. A more detailed analysis of the model is included in the Discussion.

### Constitutively active EvgS remains on in a non-acid-inducible mutant

If this hypothesis is correct, the constitutive mutations should continue to exert their effect even if EvgS can no longer detect low pH. The regions of EvgS which are required for detection of low pH have not been determined, and the actual ligand(s) detected is not known. Several other sensor kinases that detect low pH do so via residues in their periplasmic regions (Prost and Miller, 2008; Müller *et al*., 2009; Haneburger *et al*., 2011), and as EvgS contains a very large periplasmic domain (approximately 45% by mass of the total protein), it is reasonable to assume that this part of the protein plays a role in ligand detection. We tested this by constructing an in-frame deletion of most of the first predicted periplasmic domain of EvgS (domain 1). This was done by deleting a stretch of DNA between unique in-frame SwaI and PsiI sites in the gene, which removed 148 amino acids from the periplasmic domain. The resulting protein, EvgSΔSP1, was still found in the membrane fraction, as is full-length EvgS (Fig. S8). Expression of this mutant failed to complement the inability of a strain with a deletion of the chromosomal *evgS* gene to respond to low pH (Fig. S9). When the *evgS*^c^ mutations S584F or S600I were reintroduced into the gene carrying the domain 1 deletion and the activity of the p*ydeP–lacZ* fusion was measured, beta-galactosidase production at pH 7.5 was detected at levels slightly lower than those found in the EvgShis strain at pH 5.5 (Fig. S9). Thus, the deletion in domain 1 prevents activation of EvgS at a low pH, but EvgS carrying this deletion is still activated by the presence of constitutive mutations in the PAS domain. We believe this is the first direct experimental demonstration that the periplasmic domain of EvgS has a role in EvgS activation.

### EvgS is highly conserved in nearly all *E. coli* strains, but absent from the pathogenic O42 strain

We tested whether any of the residues identified in our experiments were altered in EvgS proteins from other *E. coli* or related genomes, using TBLASTN to search all complete microbial genomes for EvgS homologues. Sixty-four complete genome sequences for *E. coli* and Shigella species were compared. All Shigella genomes and all but one *E. coli* genomes (the exception being O42; see below) contained an *evgS* homologue with at least 95% identity to the gene in MG1655, with the identity in most cases being much higher. We aligned all the predicted PAS domains from these genomes. Although there were some mutations present, none were in the amino acids identified in this study, and most were conservative changes. The only substantial difference was found in *E. coli* NA114, a multi-drug-resistant UPEC strain (Avasthi *et al*., 2011) which contains a 21-amino-acid deletion in the regions corresponding to positions 656–676 in the MG1655 EvgS protein. This would be predicted to remove the penultimate beta-sheet in the PAS domain and severely disrupt the domain structure. Interestingly, this strain also lacks *ydeO*, *gadX* and *gadA* homologues, and has a truncated *gadE* gene. We therefore predict that this strain will not show a normal AR2 response to low pH.

The enteroaggregative *E. coli* strain O42 completely lacks homologues to *evgA* and *evgS*, although all the genes for AR2 are present (Chaudhuri *et al*., 2010). This is the result of a deletion event that has removed several kilobases of DNA in the region upstream of the *evgAS* operon, a region that is very variable in different strains of *E. coli*. We predicted that O42 would show poor survival of acid shock in exponential phase with or without pre-induction, and this was the case (Fig. S10(a)). In stationary phase, the strain showed some resistance to acid, presumably due to RpoS-dependent pathways, but still showed significantly lower resistance than either the MG1655 ancestor or an Stx^−^ (lacking the genes for Shiga toxins) derivative of the enterohaemorrhagic strain *E. coli* O157:H7 (Sakai) (Fig. S10(b)). Introduction of the p*evgAS* plasmid made no difference to the uninduced acid resistance of O42, but the strain now showed inducible resistance of 0.27% after 2 h at pH 2.5. Introduction of the p*evgAS^c^*N573L plasmid caused an increase in uninduced acid resistance to 56.9% (Fig. S10(c)), showing that full resistance could be restored to this strain and confirming that EvgS is essential for the detection of low pH in exponential phase growth.

## Discussion

Laboratory-based experiments that model some part of the evolutionary process have been conducted for many years and are becoming more popular as high-throughput sequencing methods make it easier to understand novel evolved phenotypes in terms of changes in genotype. Many involve growing organisms for long periods under conditions for which they are not optimally adapted, and the mutations that improve their growth are then identified and studied. These conditions have included growth in the presence of a carbon-source which model building suggested is not being used as efficiently as it could be (e.g. Ibarra *et al*., 2002; Herring *et al*., 2006), growth in the presence of a carbon source which cannot usually be utilized (e.g. Blount *et al*., 2008; 2012[Bibr b6],[Bibr b7]), and growth under nutrient limitation (e.g. Maharjan *et al*., 2010; Wang *et al*., 2010). This conceptual approach is also applied to studies on evolution of resistant organisms in people who are being treated by antibiotics (Mwangi *et al*., 2007; Howden *et al*., 2011; Lieberman *et al*., 2011). Other approaches have included gradually increasing the magnitude of the stress that is applied, to allow the accumulation of mutations which have only an incremental effect individually but which are synergistic when acting together, and allowing stepwise evolution of a new phenotype which could not be achieved by a single mutation (e.g. Rudolph *et al*., 2010; Blaby *et al*., 2012).

Our approach has been somewhat different. We used alternation between an extreme stress (pH 2.5) and normal growth conditions to select for mutations that improve survival under the extreme stress conditions. A similar approach was used to evolve populations of *E. coli* to become more resistant to the effects of ionizing radiation (Harris *et al*., 2009). Our assumption in these experiments was that the fraction of the population containing beneficial mutations will increase over time as the extreme stress is iteratively applied, and surviving mutants thus make up a larger and larger proportion of the population that is used to seed the culture grown under non-stress conditions. We then selected individual clones from highly acid-resistant populations and characterized these in detail. This particular stress was chosen as it was already known that the inducible acid stress response would provide protection against this low pH in exponential phase *E. coli*; thus, we were anticipating mutations in some aspect of the regulatory circuitry which governs this response. In the event, all the mutations were at the start of the regulatory pathway that leads to inducible resistance; we consider the likely reasons for this below. This approach should be generalizable to any organism and any stress for which there is an inducible response, and should be particularly useful as an approach to study organisms for which the nature of the inducible response is as yet poorly characterized. The approach also may be a reasonable model to represent evolving populations of *E. coli*, since as an enteric organism that has to pass the barrier of the stomach to colonize the gut, it is iteratively exposed to the extreme stress of low pH.

It is common in evolution experiments to estimate the mutation frequency, but this cannot be done here as the size of the population that passes through the bottleneck of acid stress at each step is not known, and so the number of generations cannot be reliably estimated. Nevertheless, the sequence data showed that all strains had accumulated several different unique mutations by the time the selection component of the experiment finished, over a relatively short number of generations. The fact that essentially all the acid resistance could be accounted for by transfer of the *evgS* mutants back into the parental strains shows that the other mutations had no significant contribution to this phenotype.

The EvgAS system is of interest for several reasons. It is involved in the regulation of some efflux pumps as well as the genes of the GAD or AR2 acid resistance response (Kato *et al*., 2000; Eguchi *et al*., 2003; Nishino *et al*., 2003; Ma *et al*., 2004). It was originally identified as a multicopy suppressor of a mutation of *envZ* (Utsumi *et al*., 1994) and we have shown using a systems-based approach that OmpR also plays a significant role in mediating *E. coli's* response to acid (Stincone *et al*., 2011). In addition, it has been shown in several studies to affect aspects of *E. coli* pathogenesis. For example, *evgA* overexpression was shown to repress TTSS expression in the enteropathogenic *E. coli* (EPEC) strain O127:H26, which in turn reduced actin pedestal formation when HeLa cells were exposed to this strain *in vivo* (Nadler *et al*., 2006). An *evgS* mutant was the only mutated two-component system that was found to have a significant phenotype in a study of the APEC strain O78 in turkeys, and strains with mutations in *evgA* and *evgS* were significantly attenuated in a transposon screen of O157:H7 mutants that showed reduced colonization of cattle (Dziva *et al*., 2013). As is the case with most two-component system kinases, the precise ligand that activates EvgS is not known, but the EvgAS system is activated by mild acid in a fairly narrow pH range (Burton *et al*., 2010) and also by bile salts (Yin *et al*., 2011).

The mode of action of EvgS is not known, but structural predictions show it is likely to have a large periplasmic domain, with a PAS domain in the cytoplasm immediately after the transmembrane helix. It is one of five hybrid sensor kinases in *E. coli* where activation includes an internal phosphotransfer before the phosphate is transferred to the response regulator EvgA (the other four are ArcB, BarA, TorS and YojN). Modelling of these types of kinases suggests that the internal relay may make them both more sensitive to the signal within a critical range and more robust against noise (Kim and Cho, 2006; Csikász-Nagy *et al*., 2011). Before this study, there was no information on whereabouts the signal was initially sensed on EvgS, although the large size of the periplasmic domains, and the fact that several other HKs which sense low pH do so via their periplasmic domains, made them an obvious candidate. As we show here, deletion of one of the two predicted PBP domains in EvgS leads to loss of the ability of the protein to detect low pH, but has no major effect on the activity of the constitutive mutants, consistent with the hypothesis that this part of the protein detects the initial signal; this may be pH itself, or some other ligand needed for the response.

The genes of the GAD pathway are all strongly upregulated by the constitutive mutations in EvgS, and we show here that this fully explains the acid resistance phenotype. There is considerable redundancy in the system in that upregulation either of YdeP alone or of the YdeO/GadE- regulated components of the GAD response are both sufficient to cause acid resistance at pH 2.5. This may explain why deletion of the glutamate/GABA antiporter GadC has no effect on the acid resistance phenotype at this pH, despite being required for the activity of AR2. On this basis, it might have been expected that the selection that we used would also have produced up-promoter mutants in genes such as *ydeP*, *ydeO*, or *gadE*. The fact that we did not find these may be a reflection of the relatively small number of strains studied, or the relative ease with which mutations in the PAS domain of EvgS lead to its constitutive activation, or both. Alternatively, some aspect of the selection methodology that is not captured in the assays we used may favour the survival of the *evgS* mutants.

The mutations that were found to activate EvgS in this study have not to date been observed in any sequenced genomes, implying that although many isolates of *E. coli* are highly acid resistant, constitutive activation of EvgS is not a mechanism for this. This is likely to be because this activation decreases the growth rate of strains, presumably because of the resources needed to make the many proteins which are overexpressed in the *evgS*^c^ mutant. At a neutral pH, the mutant strains are rapidly out-competed by the wild-type ancestor strain (data not shown), so unless populations of bacteria were subjected to rapidly cycling levels of pH, as in our selection experiments, the *evgS^c^* mutations would be unlikely to persist.

The role of YdeP in acid resistance has been noted before (Masuda and Church, 2003) but no explanation for it has been proposed. YdeP is annotated as a putative oxidoreductase, but in a blast search of the MG1655 genome with *ydeP* the three top hits are all to genes which components of formate dehydrogenases: *fdhF*, *fdoG*, and *fdnG*. All three of these proteins contain a selenocysteine (U) residue which is where their molybdopterin cofactors bind; YdeP has a C at this position so is unlikely to be acting as a classic formate dehydrogenase. It is intriguing to note that the operon immediately adjacent to the *evgAS* operon, which is very highly induced in the *evgS^c^* mutants, contains genes for a formyl-CoA transferase (*yfdW*) and an oxalate decarboxylase (*yfdU*). Between them these two enzymes can create a cycle where oxalate is converted to oxalate CoA by transfer of the CoA from formyl-CoA, releasing formate (Toyota *et al*., 2008). The formyl-CoA is regenerated by the action of YfdU (the most strongly induced of all the EvgS regulated genes in our array data) on oxalate-CoA, a process which uses up a proton. These two enzymes have indeed recently been shown to have a role in oxalate-induced acid resistance in *E. coli* (Fontenot *et al*., 2013). Breakdown of formate catalysed by YdeP could potentially keep this cycle turning in the absence of coupled oxalate/formate import/export, enabling *E. coli* to utilize metabolic oxalate in a novel acid resistance mechanism.

Two-component sensor kinases typically function via a change in their dimerization state, and modulation of dimerization through PAS domains has been proposed as one way in which sensor kinase activity could be modulated (reviewed in Möglich *et al*., 2009). The nature of the mutants found in this study supports a model where the PAS domain plays a key role in dimerization. Our results and modelling studies are consistent with a model where EvgS is held in a tight, inactive dimer, and where the signal caused by the interaction of one or both periplasmic domains with their activating ligand leads to a weakening of the dimer and activation of the autokinase activity of the protein. This model would explain why it was relatively easy to obtain mutations which activate EvgS, since these would all be loss-of-function mutations for dimerization, although they would lead to a gain of kinase activity in the absence of a periplasmic signal. A similar mechanism has been proposed for some of the constitutive mutations that have been studied in the *E. coli* DcuS sensor kinase, which responds to C4-dicarboxylates (Etzkorn *et al*., 2008; Monzel *et al*., 2013). A more detailed interpretation of the effects of the mutations which activate EvgS should be undertaken with care; our basic structural model will inevitably contain errors as there are uncertainties as to the appropriate sequence alignment, and variations in the position of some secondary structure elements, when comparing the experimentally solved structure s of PAS domains. The most convincing explanation is for F577S. F577 looks likely to pack into a hydrophobic pocket created by the beta sheet and the loop between strands 4 and 5, the part of the model that seems likely to be the most reliable. Thus, the F557S mutation may disrupt the hydrophobic interaction and thus the interaction between the N-helix cap of one subunit and the beta sheet domain of the other subunit. Explanations of the activation mechanisms for the other mutations are more speculative. Introduction of proline into the N-cap helix via Q574P or S582P seems most likely to effect changes by introducing a kink into the helix, thus necessitating a change in the dimer packing; however, Q574 and S582 potentially form part of a hydrogen bonding network with K579, R578, D583, and S600, which might be disrupted by mutation to proline. S600I could also disrupt such a network, with the only other obvious affect being to increase the interaction with F557, enhancing the interaction between the helix and the beta-domain, although the long exposed hydrophobic residue might also promote other hydrophobic interactions that change the favoured packing. N573L and S584F are both changes from small hydrophilic to larger hydrophobic. N573 is only peripheral to the dimer interface. It is possible to envisage small changes in the modelled structure leading to N573 interacting with the loop between beta-strands 4 and 5, and thus the effect of N573L could include promoting a hydrophobic interaction with the loop, or changing the way the coiled coil comes together by modifying the inter helix interactions that are possible. In the model S584 sits on the opposite side of the beta-sheet to F577, packing with C671 and T669, but it is also partially solvent exposed. Mutation S584F greatly increases the bulk at position 584, and it seems unlikely that the structure could accommodate this bulk without a large change in the relative orientation of the N-cap helix and beta-sheet domain. C671R might similarly be interpreted as the bulky R group disrupting the ability to pack, or alternatively having an unfavourable electrostatic interaction with K643, which is adjacent in the structure. K643E sits between E641 (circa 7 Å away), E644, (circa 10 Å away) and D671 (circa 12 Å away), D671 being in the loop between strands 4 and 5. Thus, the only obvious predictable change is a possible electrostatic repulsion that leads to remodelling of the loop. The affect of G658A is unclear; removing the flexibility associated with glycine may affect the fold, or some motion of the protein required for a conformational change. E701G is in the C-terminal helix of the PAS domain, which was not modelled. However, flexibility of G may affect helix stability, or loss of the negative charge of E may disrupt so interaction, or both. It is not possible without further experimental characterization to be sure of the exact structural modulations that occur.

Biophysical and further genetic experiments to test our model for EvgS are now in progress. In the light of our model, it is intriguing to note that a recent study shows that a truncated version of EvgS that includes the PAS domain but lacks the rest of the cytoplasmic domain shows extensive clustering in *E. coli* cells, but this is lost if the PAS domain is deleted (Sommer *et al*., 2013).

## Experimental procedures

### Strains and growth conditions

All strains and plasmids are listed in Table S1. Growth and evolution experiments were in lysogeny broth (LB; 10 g tryptone, 5 g yeast extract, 10 g NaCl per litre of medium). Acid resistance was measured by diluting cells into LB or other appropriate medium adjusted to pH 2.5 using hydrochloric acid, followed after set times by plating of 10-fold dilutions of cultures onto LB agar plates and scoring colonies after overnight growth. Unless otherwise stated, acid resistance assays, heat resistance assays and luciferase reporter assays of clonal isolates were performed in M9-cas (Burton *et al*., 2010).

### Determination of acid resistance

Acid resistance was measured by diluting cultures into LB which had been acidified to the experimental pH with hydrochloric acid, incubating them for a set length of time, and then plating them out on LB agar plates and counting colonies after overnight incubation. Viable cells at the start of the experiment were measured by dilution and plating. All experiments were performed at least three times. Resistance was expressed as a percentage survival relative to the viable colony count immediately before exposure to acid. To distinguish different acid resistance systems, resistance measurements were done on strains carrying mutations in key genes for those systems, or on minimal medium supplemented with the appropriate amino acids at 0.5 mM.

### Selection for mutations conferring acid resistance

Overnight cultures were diluted to a starting OD_600_ of 0.01 and grown with shaking at 37°C until the OD_600_ reached approximately 0.2. At this point, the cultures were serially diluted 10-fold in LB acidified at pH 2.5. After 2 h incubation at room temperature, 10 μl of each dilution was spotted onto LB agar plates which were then incubated overnight and scored for survival. At the same time, 100 μl of each dilution was added to 5 ml of fresh LB at pH 7 which was then shaken at 37°C overnight. The next day, the culture from the highest dilution that showed growth was used to repeat the process. A control culture was treated in the same way but without exposure to low pH. After 21 days, when all cultures had acquired some level of acid resistance, single colonies were isolated and checked individually for acid resistance. One colony showing high acid resistance from each culture was chosen for further characterization.

### Strain manipulations

Gene replacement mutagenesis was done as described by Datsenko and Wanner (2000). Single nucleotide chromosomal mutations were made by first removing the endogenous gene and inserting a resistance cassette. The resistance cassette was then exchanged in place of the gene containing the single nucleotide change, using gene doctoring as described by Lee *et al*. (2009). Successful mutations were screened by the loss of antibiotic resistance and sequencing.

### Plasmid construction and site-directed mutagenesis

Plasmids pEvgAS^c^N573L and pEvgAS^c^S584F were constructed by amplifying the *evgAS* operon, including its promoter, from the genomic DNA of the relevant evolved strains. The primers used were (upstream of *evgA*) 5′-CCGATGGATCCACTACCTGAGACTTGTGCAG and 5′-CCGATGCTAGCCCACATTTGAACATTGTGGG, which respectively contain novel BamHI and NheI restriction sites. Plasmid pEvgA was constructed using the same upstream primer, and 5′-GGTTAGCTAGCTTAGCCGATTTTGTTACG, which contains an NheI site. PCR was performed using Phusion High-Fidelty Master Mix (Finnzymes, Finland), and PCR fragments were purified using the Qiaquick PCR purification kit (Qiagen, UK). Fragments were ligated with pZC320 plasmid DNA (Shi and Biek, 1995) digested with BamHI and NheI, and candidate plasmids were confirmed by sequencing the PCR insert. The plasmid pydeP-tet was constructed by ligating an NcoI-digested PCR fragment carrying the tetracycline-resistance gene from plasmid pBR322, amplified using the primers 5′-GACAGCTTACCATGGATAAGC and 5′-CAGTTCTCCCCATGGATTGATTG, into the plasmid pydeP-lux (Burton *et al*., 2010), digested with NcoI. Plasmids containing fragments in the correct orientation were identified by DNA sequencing. Site-directed mutagenesis was done using an Agilent QuikChange mutagenesis kit, as recommended by the manufacturer.

### Whole-genome sequencing and analysis

Whole genomic DNA for each strain (including the ancestor MG1655 strain) was analysed on the Illumina GA2 sequencing platform at the Washington University Genome Center, generating 35 base paired-end reads. The resulting FASTQ files were analysed using the xBASE-NG facility against the *E. coli* K-12 MG1655 reference sequence. xBASE-NG employs MAQ for alignment and SNP calling (Chaudhuri *et al*., 2008; Li *et al*., 2008). All *evgS* mutations were confirmed by Sanger dideoxy terminator sequencing.

### Luciferase assays

Luciferase assays were performed as described in Burton *et al*. (2010), except that promoter activity was measured at a single time point. Strains to be assayed were grown in 96-well polypropylene plates sealed with a gas-permeable membrane. At OD_600_ 0.2 the cultures were transferred to an opaque white walled and bottomed 96-well polypropylene plate to measure the luciferase activity with an automated plate reader (Fluroskan Ascent, Thermo Scientific, Basingstoke, UK).

### Array methods

EvgS^c^ (*n* = 2) and wild-type (*n* = 2) cultures were used for microarray profiling. RNA was isolated from cells using the Qiagen RNeasy kit (Qiagen, USA) according to the manufacturers' instructions. RNA purity and quality were checked using a Nanodrop spectrophotometer (Thermo Scientific, USA) and Agilent Bioanalyser system respectively. The 260/280 nm absorbance ratio was between 1.8 and 2.1 for all samples and the average RIN score was > 8. Fifty nanograms of input RNA was labelled with Cy3 dye using an Agilent Low-Input Quick Amp kit (Agilent, USA) with random primers according to the manufacturer's protocol. Six hundred nanograms of labelled cDNA was hybridized overnight to Agilent *E. coli* Gene Expression Microarrays then immediately washed and scanned using an Aglient SureScan microarray scanner in accordance with the recommended procedure.

Array data were normalized using quantile normalization (Bolstad *et al*., 2003). Fold change ratios were calculated between all wild-type and EvgS^c^ sample pairs. Differentially expressed genes were identified using the fold change ratios by applying a one sample *t*-test followed by Benjamini-Hochberg FDR correction (Benjamini and Hochberg, 1995). Genes with a false discovery rate (FR) < 1% and an average log_2_-fold change of > 2 were defined as differentially expressed.

### Structural modelling of the EvgS PAS domain

A PSI Blast search via the NCBI web interface of the non-redundant database returned 3000 hits after three iterations. The Position-Specific Scoring Matrix (PSSM) produced by this search was used for a PSI Blast search of the PDB. The PSSM represents the likely amino acid at each position in a given sequence type and thus allows the detection of remote homologues. Top hits were PDB codes 3LYX, 3EWK, 3BWL and 3A0R. PDB ID 4I5S was also subsequently added to our sequence alignment for reasons described later in this section. A sequence alignment of the five PAS domain templates was created manually using Seaview (Gouy *et al*., 2010), guided by the secondary structure of these templates, and a comparison of their overlaid 3D structures was viewed in Pymol. Secondary structures of the templates were determined from their 3D structure by the software DSSP, which recognizes secondary structure by backbone hydrogen bond patterns (Kabsch and Sander, 1983). The secondary structure of the EvgS PAS domain predicted by PsiPred (McGuffin *et al*., 2000) guided the sequence alignment of EvgS with respect to the known structures. Initial comparative models based on chain A of 3A0R were produced using Modeller 9.12 (Sali and Blundell, 1993). PIER analysis, which predicts the site of likely protein interactions via the physicochemical properties of the atoms of the surface residues, was conducted for the best five models [as judged by GA_341_ score (Melo and Sali, 2007)]. GA_341_ is a genetic algorithm derived function built into the Modeller software that combines the percentage sequence identity between templates and model sequence, a statistical potential dependent on inter-residue distance and solvent accessibility, and the compactness of the final model. The average over these five models was calculated for each residue and is displayed in Fig. S7. A dimer form of the EvgS PAS domain was modelled using Modeller 9.2. Two hundred and fifty structures were produced using 3A0R and 2BWL together as templates, and 250 structures were produced using 3A0R, 2BWL and 4I5S together as templates. The models produced with 3A0R and 2BWL as templates had the highest DOPE scores (Shen and Sali, 2006; DOPE is a statistical potential built into Modeller that is designed to determine good quality structural models). These templates also produced models with the best results in a Whatcheck analysis (Hooft *et al*., 1996), which assesses properties such as Ramachandran plot and side-chain packing. The model with highest DOPE score is shown in Fig. 6.

### Online data

The microarray data are on GEO with Accession No. GSE56833
