# Supplementary material for: Characterization of mutations in the PAS domain of the EvgS sensor kinase selected by laboratory evolution for acid resistance in Escherichia coli
Source: Mol Microbiol. 2014 Jul 24;93(5):911–27. doi: 10.1111/mmi.12704 (PMC4283999; doi:10.1111/mmi.12704)
Supplement: Supplementary file 1 — Supporting Information [file mmi0093-0911-sd1.pdf]

Supplementary material

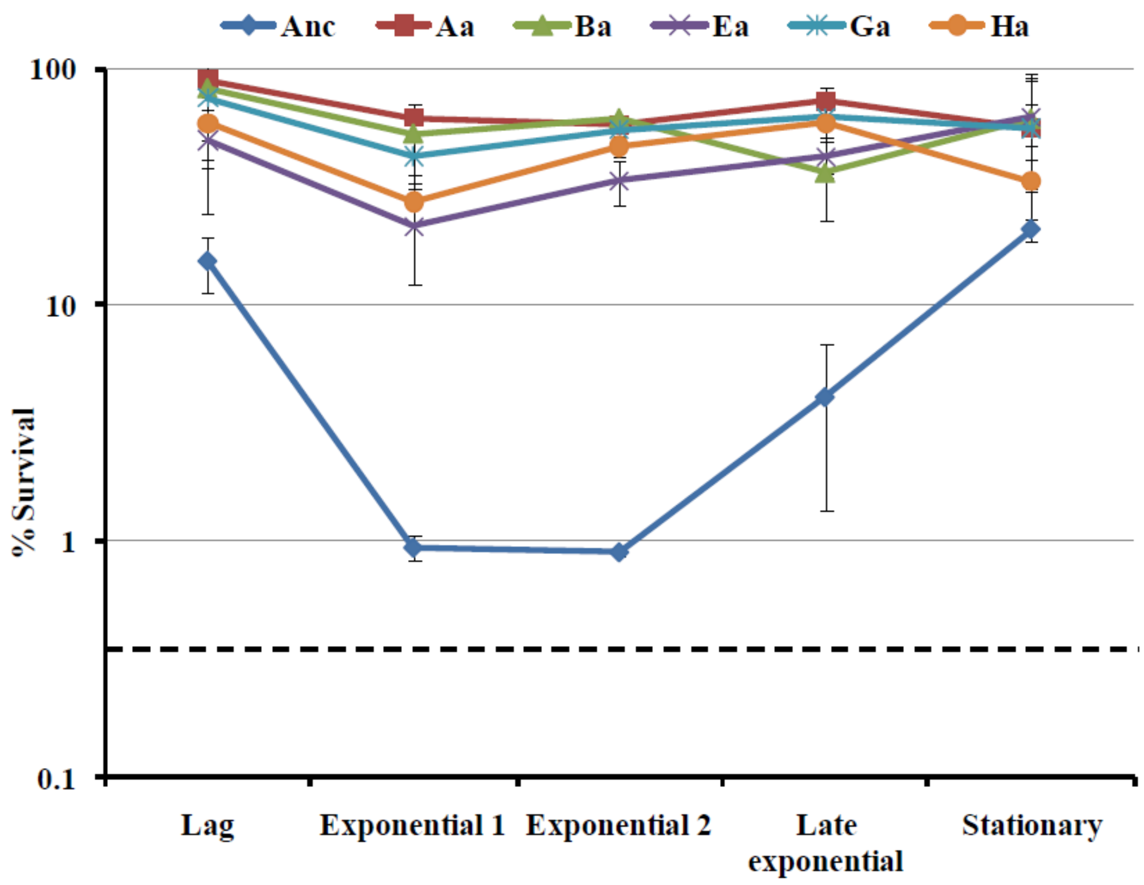

Figure S1. Acid resistant mutants show enhanced resistance at all stages of growth. Survival of acid resistant colonies isolated from each evolving cell line (Aa-Ha), and the MG1655 ancestor strain (here labelled as "Anc"), after 2 hours at pH 2.5, at different stages of growth. Percentage survival was calculated by dividing the number of colonies after 2 hours in pH 2.5 by the number of colonies at time zero at pH 7. The error bars are the standard deviations of three independent biological replicates.

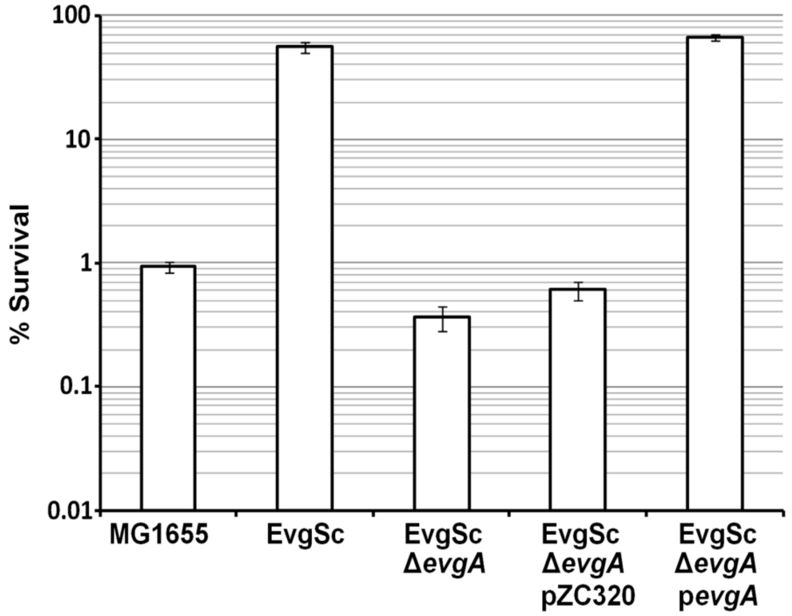

Figure S2. The acid resistance phenotype caused by the presence of an EvgS^{c} mutation requires the presence of EvgA. Levels of acid resistance were measured in the strains shown; in all cases the *evgS*^{c} mutation used was evgS-G658A. Deletion of *evgA* causes complete loss of *evgS*^{c}-induced acid resistance, and this is fully restored by a plasmid expressing *evgA* (p*evgA*) but not by vector alone (pZC320).

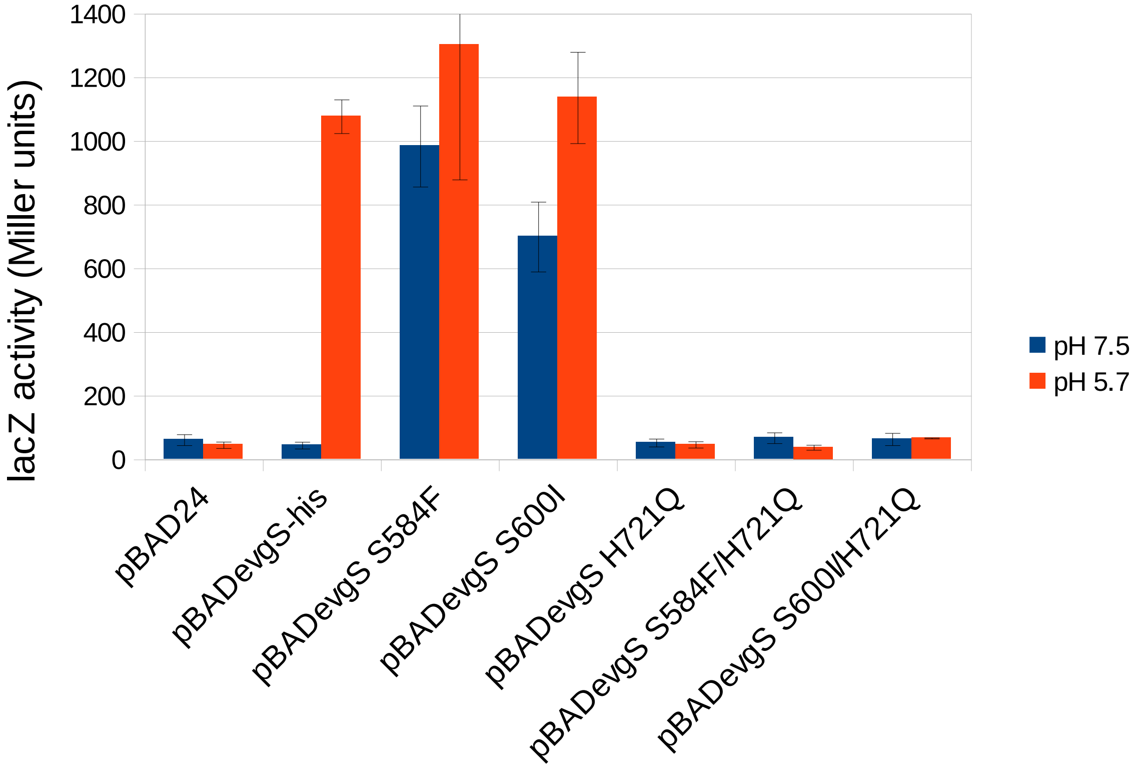

Figure S3. Activity of EvgS and EvgS^{c} is abolished by mutation of H721 to Q. LacZ activity was measured from a chromosomal p*ydeP-lacZ* fusion in the presence of the plasmids indicated, at either pH 7.5 or pH 5.5. All mutants are in evgS-his expressed in the plasmid pBAD24. Data are means and standard deviations of three independent biological replicates.

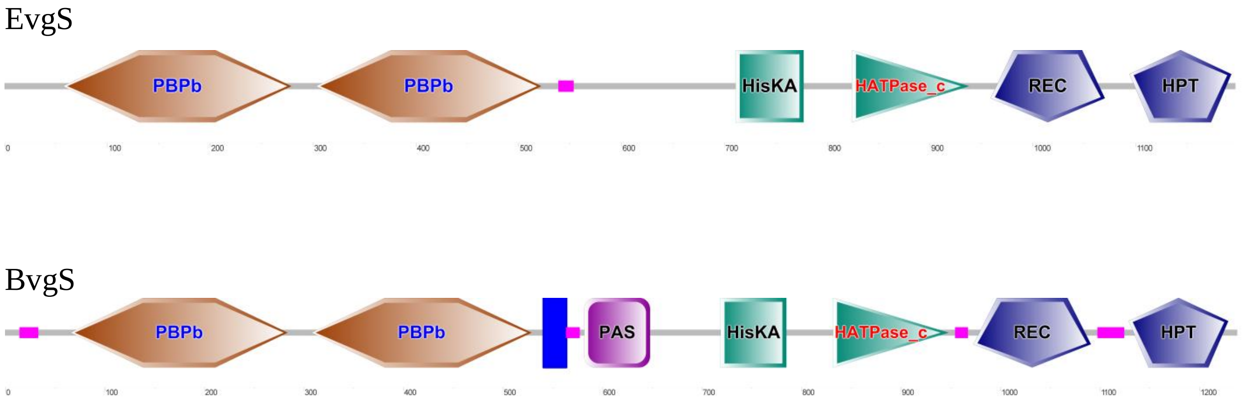

Figure S4. SMART prediction of domain structure in EvgS and BvgS. Although SMART does not identify a PAS domain for EvgS this is likely to be due to the threshold for the HMM in SMART being set to avoid false positives. A PSI-Blast search against the PDB with the EvgS putative PAS domain returns only PAS domains as significant hits.

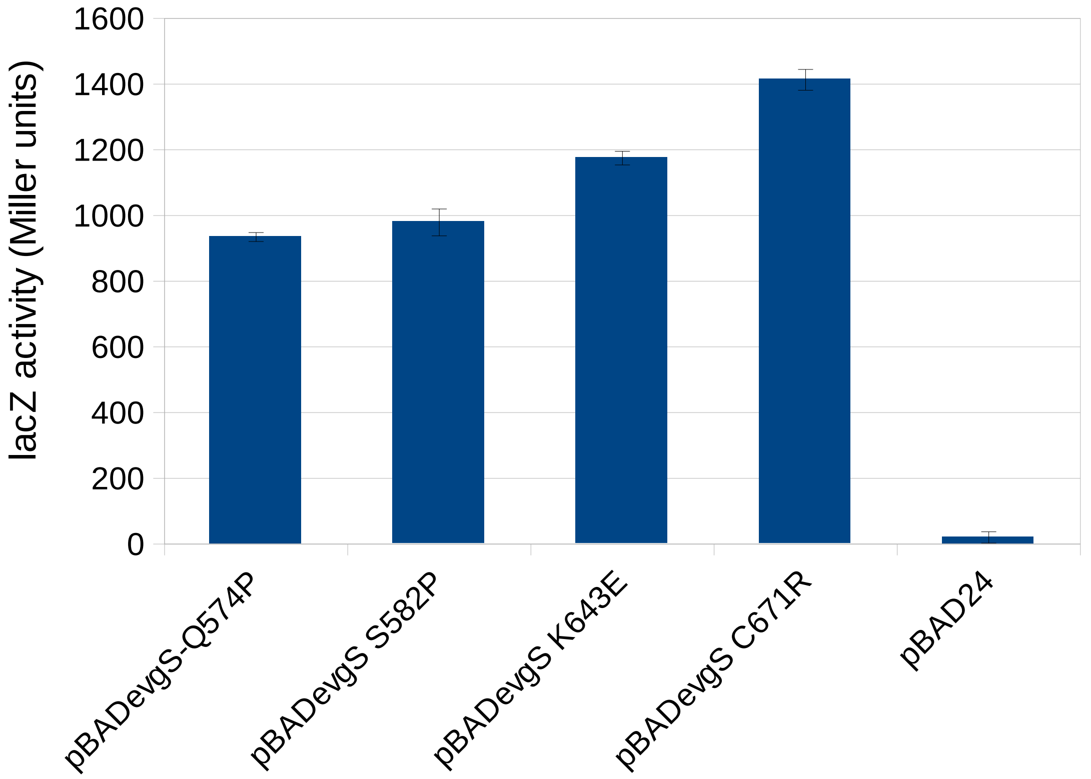

Figure S5. Activity of p*ydeP-lacZ* fusion in strains carrying pBAD-*evgS-his* plasmid with mutations in the PAS domain, selected for using the p*ydeP-tet* fusion plasmid. All cultures were grown at pH 7. Data shown are means and standard deviations of three independent experiments.

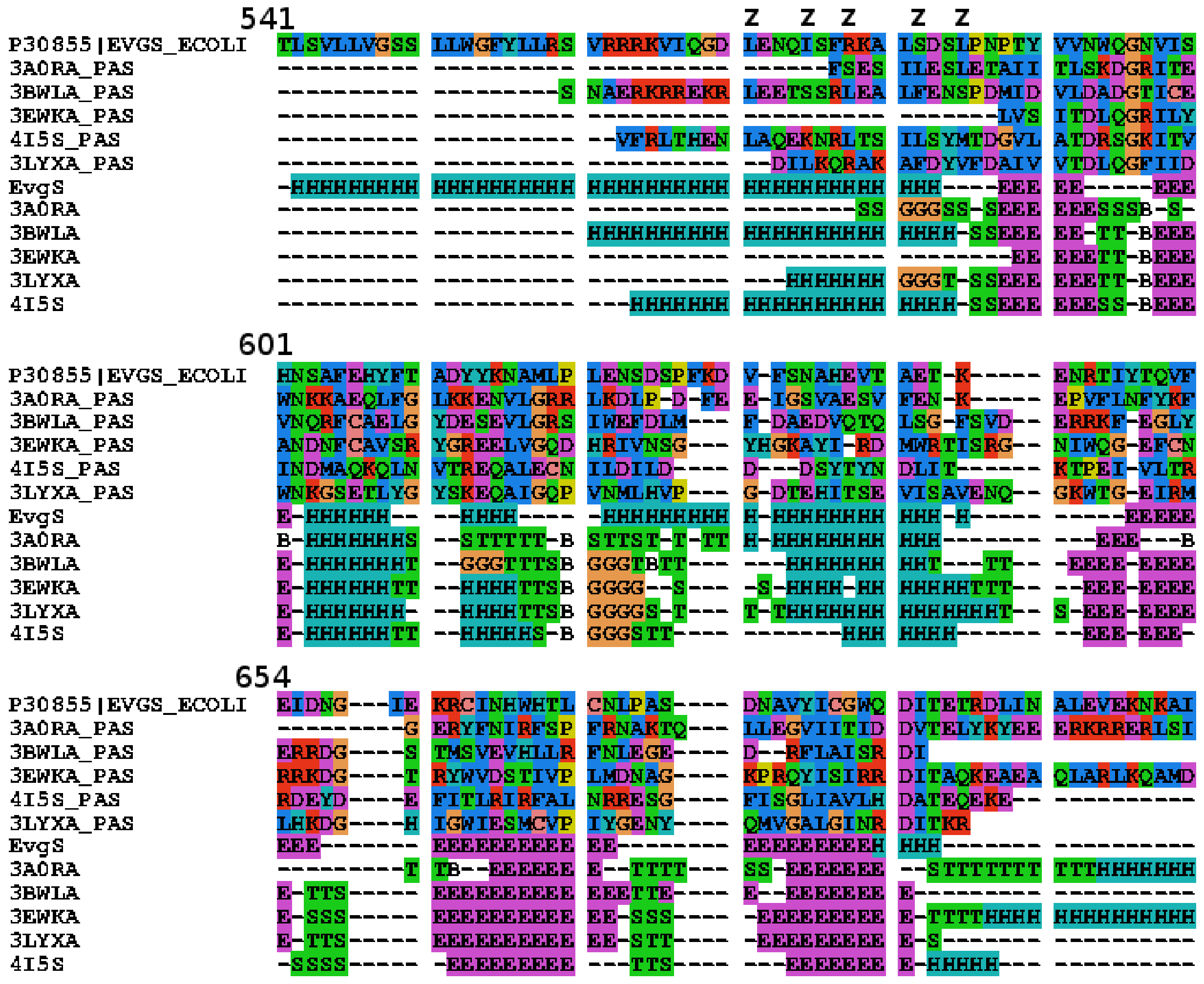

Figure S6. Sequence alignment used for comparative modelling of the EvgS structure. The figure shows sequence alignment of 5 structural examples of PAS domains along with the PAS domain of EvgS. The PDB codes of each structure are given at the start of each row, excepting EvgS, which is denoted by its Uniprot identifier. The first five rows give the amino acid sequence of the PAS domains, the lower five rows indicate the secondary structure predicted by psi-pred for EvgS and the observed secondary structure of the structural examples, as determined by DSSP; H indicates an alpha helix, E a beta strand, G a 3/10 helix, B a beta-bridge, T a turn and S a bend, as per the convention for DSSP (Kabsch et al., 1983). Z above the alignment indicates key residues for the coiled coil interaction seen in the dimeric structures formed by 3BWL and 4I5S. See the main text for further details

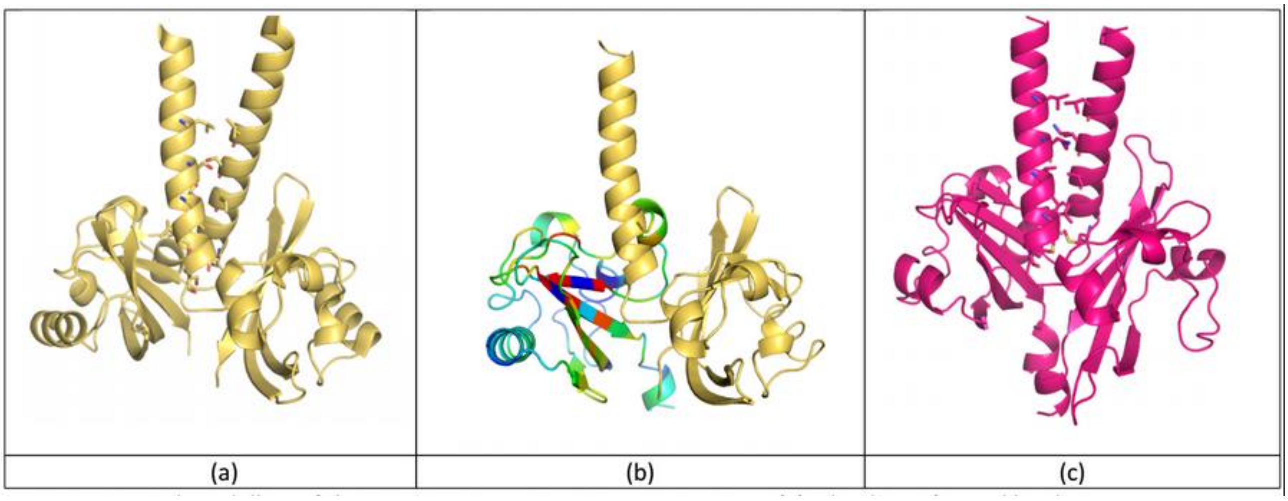

Figure S7. PIER analysis indicates a protein interaction site consistent with dimer formation. (a). The dimer formed by chains A and B in the crystal structure of the PAS domain dimer of HTR-like protein from *Haloarcula marismortui (*3BWL). The protein backbone is represented by a ribbon, with the side chains of key residues in coiled-coil formation shown as sticks. (b) A Model of EvgS using template structure 3A0R, displayed in the position of chain A of the 3BWL structure, alongside the chain B of 3BWL. The EvgS model is coloured through the spectrum by PIER values where red indicates a high PIER value, (i.e. predicted interface residue), and blue represents a low PIER value, the monomer from the 3BWL dimer structure is straw coloured. The red/orange residues on the beta-sheet have side chains that point upwards, i.e. towards the dimer interface shown here. The PIER values displayed are the average value for each residue averaged over the five best models selected from the 250 monomeric models produced by Modeller. An average was taken to minimize artefactual errors arising from the variability of side-chain positioning between models. (c) PAS domain from the VicK protein of *S. mutans*, (PDB ID 4I5S).

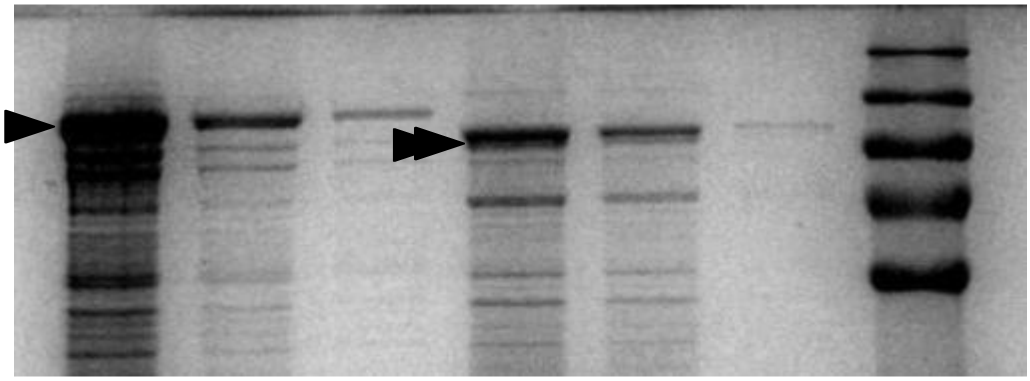

Figure S8. SDS-PAGE of membrane fractions of *E. coli* expressing either pBAD-EvgS-his or pBAD-EvgSΔSP. To prepare membrane fractions, overnight cultures of cells containing either pBAD-EvgS-his or pBAD-EvgSΔSP were grown in 100 ml YT medium containing arabinose (0.2% w/v) to an OD600 of 1. Cells were collected by centrifugation, resuspended in 16 ml and then 8 ml buffer M (50 mM TEA pH 7.5; 1 mM DTT, 1 mM EDTA, 1 Roche protease inhibitor tablet per 100 ml) and then lysed by sonication. The sonicate was spun for 90 minutes at 20,000 rpm in a Beckman JA20 rotor, and the resulting pellet resuspended in 4 ml buffer M. This was respun under the same conditions for 1 hour, and the pellet was resuspended in 1.4 ml buffer M. Samples were loaded as follows: tracks 1-: extract from pBAD-EvgS-his expressing cells; tracks 4-7: extract from pBAD-EvgS-his expressing cells (sample sizes of 10 μl, 5 μl and 1 μl respectively). Track 7: molecular weight markers (200kDa, 150kDa, 100kDa, 80kDa, 60kDa). Identity of protein bands was confirmed by tandem MS/MS analysis. The EvgS-his and EvgSΔSP are marked with single and double arrows respectively.

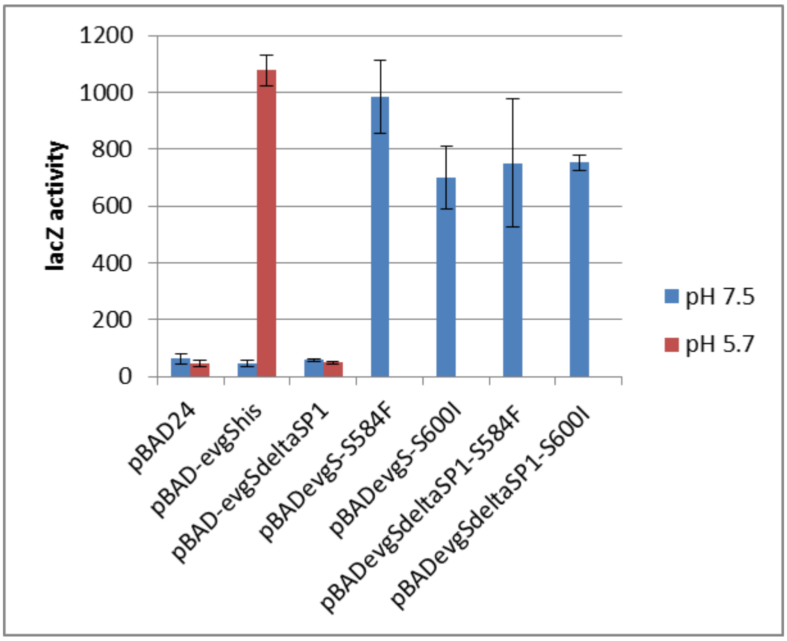

Figure S9. The deletion of domain 1 in EvgS leads to loss of ability to respond to low pH, but *evgS*[c] mutations still constitutively up-regulate *ydeP* activity. Activity of *ydeP-lacZ* fusion in strains carrying different pBAD-*evgS-his* plasmids. Data shown are means and standard deviations of three independent experiments. Experiments at pH 5.7 were only performed for pBAD24, pBAD-evgShis, pBAD-evgSdeltaSP1.

A

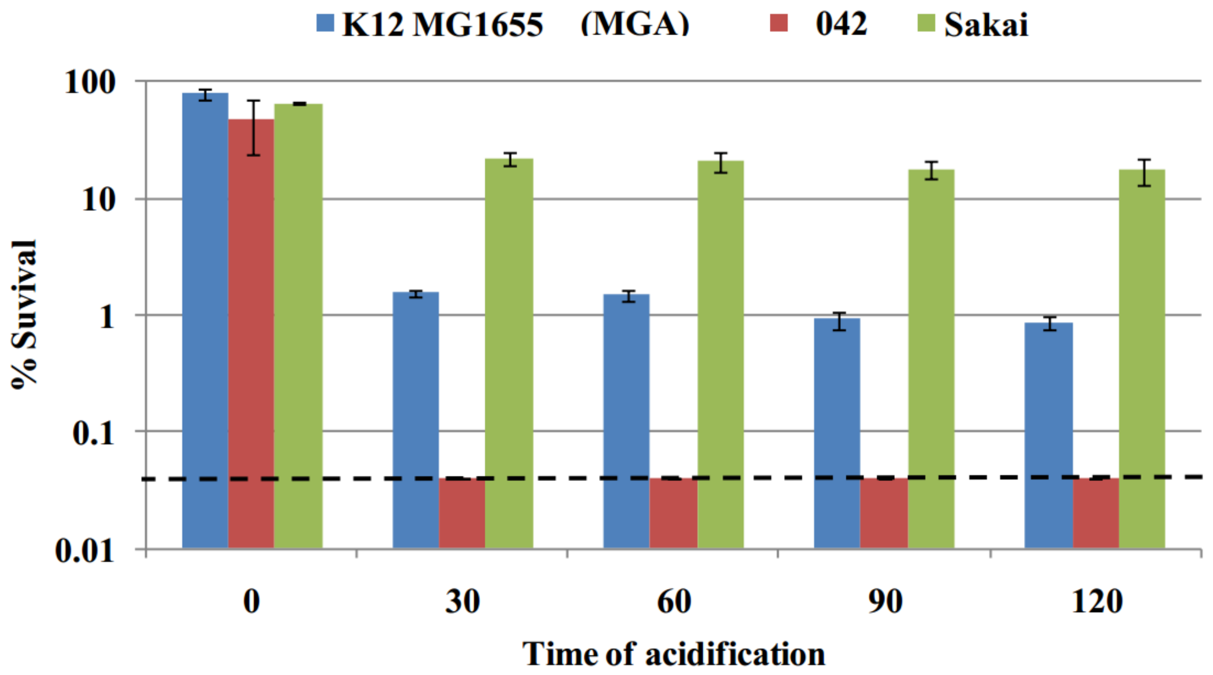
K12 MG1655 (MGA)
042
Sakai
100
10
1
0.1
0.01
% Suvival
0
30
60
90
120
Time of acidification

B

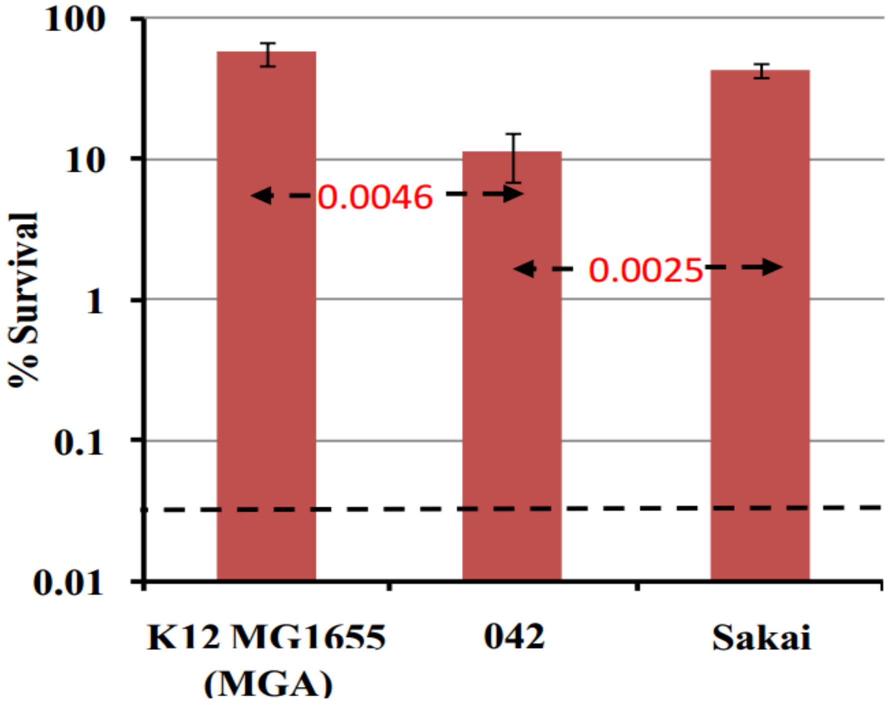
100
10
1
0.1
0.01
% Survival
0.0046
0.0025
K12 MG1655 (MGA)
042
Sakai

C

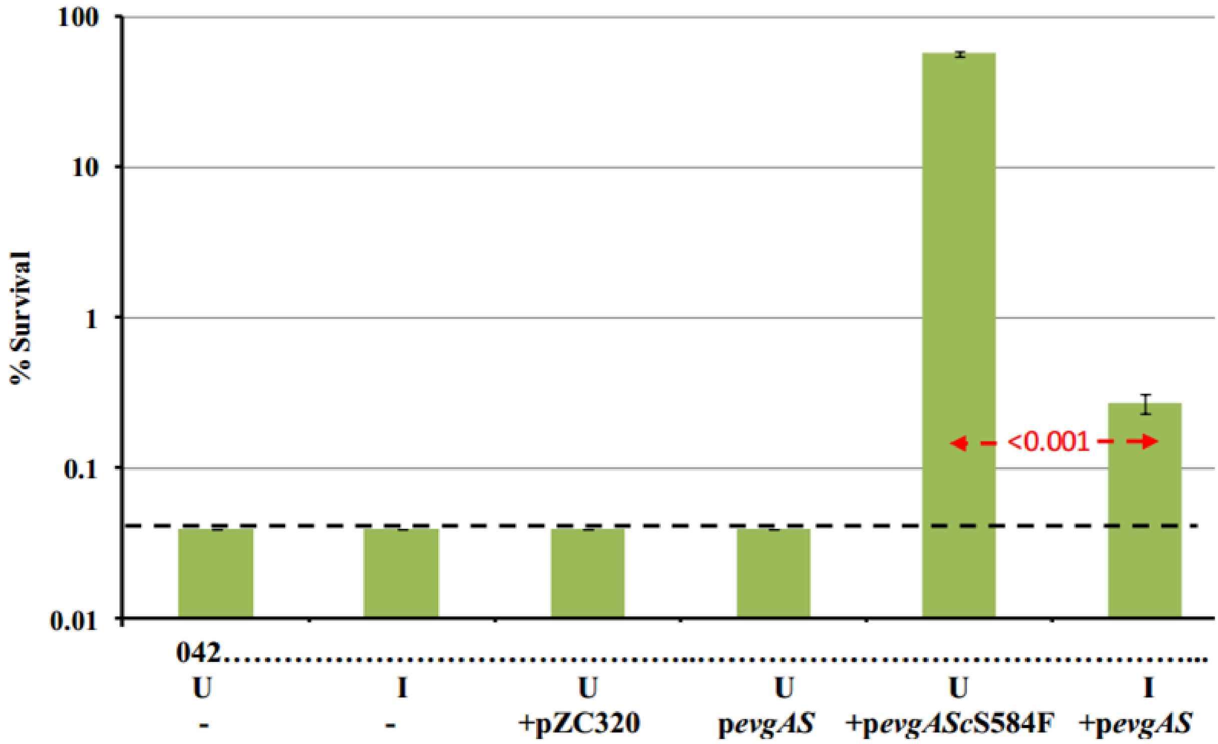

Figure S10. Acid resistance of *E. coli* O42, compared to MG1655 and O157:H7 (Sakai). (A) Survival of exponential phase MG1655 (labelled MGA), O42 (labelled O42), and O157:H7 (Sakai) (labelled Sakai) at different times after acidification to pH 2.5. (B) Survival of the same three strains grown to stationary phase and exposed for 2 hours in M9-cas at pH 2.5. (C) Acid resistance of O42 in the presence of plasmids containing wild-type *evgAS* or evgAS^{c}S584F. pZC320 is the vector only control. U = uninduced, I = induced. The dotted horizontal line in each case shows the minimum survival detectable by this assay. Values in red are p-values for T-tests, and the arrows show which sets of data were compared. All assays were repeated three times; error bars are standard deviations.

Table S1. Strains and plasmids used in this study.

**Strain name**	**Relevant genotype**	**Reference/source**
MG1655	*ilvG*⁻ *rfb-50 rph-1*	Bachman, 1972
MG1655-Aa	Acid resistant clone from long term selection experiment	This work
MG1655-Ba	Acid resistant clone from long term selection experiment	This work
MG1655-Ea	Acid resistant clone from long term selection experiment	This work
MG1655-Ga	Acid resistant clone from long term selection experiment	This work
MG1655-Ha	Acid resistant clone from long term selection experiment	This work
MG1655 *evgS*-G658A	MG1655 containing evgS-G658A mutation	This work
MG1655 *evgS*-S600I	MG1655 containing evgS-S600I mutation	This work
MG1655 *evgS*-G658A Δ*evgA*	MG1655 containing evgS-G658A mutation with *evgA* gene deleted	This work
MG1655 Δ*evgS* p*ydeP-lacZ*	MG1655 with *evgS* gene deleted and with chromosomal fusion of *ydeP* promoter to *lacZ*	Utsumi and Eguchi, unpublished
E. coli O157:H7 Sakai Stx⁻	Wild type O157:H7 (Sakai) with *stx1* and *stx2* genes deleted	Lee *et al.*, 2009
E. coli O42	EAEC *E. coli*	Chaudhuri *et al.*, 2010
E. coli XL1-red	Mutator strain	Agilent technologies
**Plasmid name**	**Features**	**Reference/ source**
pBAD-EvgS-his	*evgS* gene with a 6-his C-terminal extension cloned between *Nco*I and *Pme*I sites of pBAD-TOPO	Utsumi/Eguchi, Kinki Univeristy
pBAD-EvgS-H721Q	pBAD-EvgS-his carrying with *evgS* H721Q mutation	This work
pBAD-EvgS-S584F	pBAD-EvgS-his carrying with *evgS* S584F mutation	This work
pBAD-EvgS-S584F/H721Q	pBAD-EvgS-his carrying with *evgS* S584F and H721Q mutations	This work
pBAD-EvgS-S600I	pBAD-EvgS-his carrying with *evgS* S600I mutation	This work
pBAD-EvgS-S600I/H721Q	pBAD-EvgS-his carrying with *evgS* S600I and H721Q mutations	This work
pBAD-EvgS-ΔSP	pBAD-EvgS-his with sequences for domain 1 removed by deleting a unique *Swa*I-*Psi*I fragment	This work
pEvgA	pZC320 with *evgA* gene ligated between *Bam*HI and *Nhe*I sites	This work

pEvgAS^{c}N573L	pZC320 with *evgAS* operon ligated between *Bam*HI and *Nhe*I sites, *evgS* carrying N573L mutation	This work
pEvgAS^{c}S584F	pZC320 with *evgAS* operon ligated between *Bam*HI and *Nhe*I sites, *evgS* carrying S584F mutation	This work
pydeP-lux	*ydeP* promoter upstream of bacterial *lux* operon in plasmid pLux	Burton *et al*., 2010
pydeP-tet	pydeP-lux with *tet*^{R} gene downstream of p*ydeP* promoter	This work
pZC320	Low copy number cloning vector, *amp*^{R}	Shi and Biek, 1995

Table S2. Summary of the sequence changes seen in each evolved strain in comparison to the ancestor MG1655 strain.

Strain	Gene	Annotation	Mutation	Base change; AA change	Position
Aa	*evgS*	Hybrid sensory histidine kinase in two component regulatory system with EvgA	missense	TCC>TTC; Ser>Phe	2484146
	arcB	Hybrid sensory histidine kinase in two component regulatory system with ArcA	missense	CGT>CTT; Arg>Leu	3348730
	eptB	Predicted metal dependent hydrolase	missense	TAC>TCC; Tyr>Ser	3708410
Ba	*hofB*	Conserved protein with nucleoside triphosphate hydrolase domain	silent	GTG>GTA; Val>Val	116446
	ybfQ	none	missense	GAA>TAA; Glu>stop	735860
	poxB	Pyruvate dehydrogenase (pyruvate oxidase), thiamin-dependent, FAD-binding	missense	ACC>CCC; Thr>Pro	901034
	narZ	Nitrate reductase 2 (NRZ), alpha subunit	missense	GTG>GGG; Val>Gly	1537952
	yfcU	none	missense	AGT>AGG; Arg>Ser	2451224
	evgS	Hybrid sensory histidine kinase in two component regulatory system with EvgA	missense	AAC>AAA; Asn>Lys	2484114
	gltX	Glutamyl-tRNA synthetase	missense	CAC>CCC; His>Pro	2518315
	ypjC	none	missense	TGC>GGC; Cys>Gly	2781995
	ggt	gamma-glutamyltranspeptidase	missense	ACT>CCT; Thr>Pro	3584678
	rpoC	RNA polymerase, beta prime subunit	missense	CCG>TCG; Pro>Ser	4184630
Ea	*dcp*	Dipeptiyl carboxypeptidase II	missense	GAG>GGG; Glu>Gly	1624365
	evgS	Hybrid sensory histidine kinase in two component regulatory system with EvgA	missense	GGC>GCC; Gly>Ala	2484368
	iscR	DNA-binding transciptional repressor	missense	TAT>CAT; Tyr>His	2660033
	yjjA	Conserved protein	missense	GTA>ATA; Val>Ile	4597945
Ga	*ylbE*	Predicted protein, C terminal fragment (pseudogene)	silent	GAA>GAG; Glu>Glu	547694
	evgS	Hybrid sensory histidine kinase in two component regulatory system with EvgA	missense	AGT>ATT; Ser>Ile	2484194
	fimD	Outer membrane usher protein, type 1 fimbrial synthesis	silent	GCC>GCT; Ala>Ala	4543253

Table S3. Gene expression data for figures 3 and 4. Figures are means and standard deviations (minimum of three determinations) of luciferase levels with the strain or relevant plasmid carrying *evgS*[c] shown in the left hand column, and the promoter probe plasmid shown in the top row.

Strain	Statistic	pLUXacpp	pLUXb1500p	pLUXevgAp	pLUXgadAp	pLUXgadBp	pLUXgadEp	pLUXgadWp	pLUXgadXp	pLUXgadYp	pLUXhdeAp	pLUXhdeDp	pLUXmgtAp	pLUXslpp	pLUXydePp
MG1655	Average	25.3	0.0	4.0	0.1	0.1	0.2	1.0	1.6	1.3	0.9	0.2	0.6	0.2	0.0
	St Dev	2.7	0.0	0.6	0.0	0.0	0.0	0.1	0.2	0.2	0.3	0.1	0.1	0.0	0.0
Aa	Average	20.5	46.2	3.5	27.2	20.7	89.3	5.4	5.8	91.2	80.7	5.7	11.8	27.8	78.5
	St Dev	1.9	1.4	0.1	3.3	1.6	1.2	0.3	0.1	6.2	4.4	0.6	1.2	1.7	1.8
Ba	Average	23.4	36.2	3.5	10.6	12.0	67.5	7.4	3.5	85.8	75.7	4.1	11.3	16.4	71.4
	St Dev	0.3	2.0	0.3	0.6	0.9	2.2	0.5	0.7	3.8	2.4	0.2	0.2	0.9	3.3
Ea	Average	33.3	49.2	4.5	25.9	25.9	101.6	8.8	5.4	99.2	85.4	10.4	19.5	40.2	97.9
	St Dev	1.7	3.9	0.4	2.3	2.7	3.4	0.4	0.4	3.7	1.7	0.4	0.3	4.2	10.3
Ga	Average	23.4	22.3	4.1	9.2	11.0	55.8	5.0	4.0	55.0	53.3	6.4	8.1	12.1	37.2
	St Dev	2.2	1.8	0.5	2.3	2.8	3.7	0.4	0.3	9.9	8.4	1.0	0.3	0.8	0.8
Ha	Average	24.3	10.6	3.7	20.3	6.8	35.7	5.1	4.6	41.7	51.3	4.9	6.2	8.6	21.9
	St Dev	1.6	0.2	0.3	2.1	0.2	1.0	0.4	0.5	3.5	1.7	0.2	0.1	0.6	1.1
+pevgAS	Average	24.9	0.4	5.2	0.0	0.1	0.1	0.9	1.3	1.5	0.8	0.1	0.7	0.2	0.0
	St Dev	2.3	0.5	1.0	0.0	0.0	0.0	0.2	0.1	0.3	0.1	0.0	0.1	0.0	0.0
+pevgAScS584F	Average	24.8	43.0	3.8	25.5	26.3	94.1	8.1	8.7	103.6	77.9	6.7	14.6	42.7	91.5
	St Dev	2.6	1.0	0.1	2.1	1.4	12.6	0.8	0.7	11.9	8.5	0.3	0.3	0.9	3.2
+pevgAScN573L	Average	23.5	34.8	3.9	18.9	25.4	80.3	6.5	6.6	69.9	58.3	6.5	11.4	25.2	61.6
	St Dev	1.3	0.7	0.2	0.8	3.2	4.1	0.4	0.4	1.1	4.8	0.2	0.3	1.2	5.1
evgScG658A	Average	24.8	6.2	5.2	2.3	2.8	16.5	3.7	2.7	17.3	18.7	4.1	4.0	4.1	10.5
	St Dev	0.4	0.1	0.3	0.7	0.6	1.2	0.2	0.1	1.1	2.4	0.1	0.1	0.0	0.5
evgScS600I	Average	23.3	18.5	4.7	6.1	6.4	40.6	4.1	4.5	43.1	35.4	5.5	7.6	10.9	32.1
	St Dev	1.5	1.8	0.5	1.1	0.7	8.8	0.4	0.7	4.5	1.8	0.8	0.3	1.4	0.4

Table S4. Summary of array results from evgS^{c} mutant S600I. Genes with a > 4-fold increase in their expression levels compared to wild type, and a P-value of <0.05, are shown. Genes known to be involved in acid resistance are highlighted in green, those involved in efflux are highlighted in yellow.

name	EvgS^{c} - WT log 2 fold change	EvgS^{c} - WT P Value	b number	annotation
frc	8.37	0.0015	b2374	formyl-CoA transferase, NAD(P)-binding
hdeA	7.99	0.0027	b3510	stress response protein acid-resistance protein
yegR	7.75	0.0023	b2085	predicted protein
hdeB	7.72	0.0039	b3509	acid-resistance protein
gadE	7.18	0.0013	b3512	DNA-binding transcriptional activator
yfdX	6.97	0.0027	b2375	predicted protein
slp	6.69	0.0061	b3506	outer membrane lipoprotein
oxc	6.59	0.0021	b2373	predicted oxalyl-CoA decarboxylase
ansB	6.20	0.0151	b2957	periplasmic L-asparaginase II
appB	6.17	0.0037	b0979	probable third cytochrome oxidase subunit II
yfdV	6.16	0.0029	b2372	predicted transporter
dcuC	6.04	0.0097	b0621	c4-dicarboxylate anaerobic carrier DcuC
ydeP	6.02	0.0039	b1501	predicted oxidoreductase
gadB	6.02	0.0008	b1493	glutamate decarboxylase B, PLP-dependent
appC	5.98	0.0045	b0978	probable third cytochrome oxidase subunit I
hdeD	5.73	0.0026	b3511	acid-resistance membrane protein
raiA	5.73	0.0102	b2597	cold shock protein associated with 30S ribosomal subunit
glpB	5.71	0.0041	b2242	sn-glycerol-3-phosphate dehydrogenase (anaerobic), membrane anchor subunit
narG	5.70	0.0309	b1224	nitrate reductase 1 alpha subunit
glpC	5.66	0.0114	b2243	sn-glycerol-3-phosphate dehydrogenase (anaerobic), small subunit
narK	5.59	0.0176	b1223	nitrite extrusion protein
ydeO	5.55	0.0043	b1499	predicted DNA-binding transcriptional acfivator
yjjI	5.49	0.0173	b4380	conserved protein
nrfA	5.32	0.0061	b4070	nitrite reductase, formate-dependent, cytochrome
nirD	5.25	0.0119	b3366	nitrite reductase, NAD(P)H-binding, small subunit
glpA	5.20	0.0133	b2241	sn-glycerol-3-phosphate dehydrogenase (anaerobic), large subunit, FAD/NAD(P)-binding
malK	5.16	0.0112	b4035	fused maltose transport subunit, ATP-binding component of ABC superfamily -!- regulatory protein
gadA	5.15	0.0012	b3517	glutamate decarboxylase A, PLP-dependent
tdcA	5.15	0.0270	b3118	DNA-binding transcriptional activator
emrK	5.03	0.0085	b2368	EmrKY-TolC multidrug resistance efflux pump, membrane fusion protein component
nirB	5.01	0.0095	b3365	nitrite reductase, large subunit, NAD(P)H-binding
gatB	4.95	0.0142	b2093	galactitol-specific enzyme IIB component of PTS
gatA	4.89	0.0107	b2094	galactitol-specific enzyme IIA component of PTS
adiC	4.86	0.0043	b4115	arginine:agmatin
mdtE	4.83	0.0031	b3513	multidrug resistance efflux transporter

appA	4.80	0.0034	b0980	phosphoanhydride phosphorylase
gatZ	4.73	0.0091	b2095	D-tagatose 1,6-bisphosphate aldolase 2, subunit
nrfB	4.60	0.0085	b4071	nitrite reductase, formate-dependent, penta-heme cytochrome c
yjjW	4.38	0.0208	b4379	predicted pyruvate formate lyase activating enzyme
lamB	4.32	0.0110	b4036	maltose outer membrane porin (maltoporin)
hyaA	4.27	0.0008	b0972	hydrogenase-1 small subunit
mdtF	4.24	0.0008	b3514	multidrug transporter, RpoS-dependent
flu	4.24	0.0019	b2000	CP4-44 prophage; antigen 43 (Ag43) phase-variable biofilm formation autotransporter
gatC	4.22	0.0403	b2092	galactitol-specific enzyme IIC component of PTS
frdB	4.20	0.0037	b4153	fumarate reductase (anaerobic), Fe-S subunit
ycbJ	4.13	0.0049	b0919	conserved protein
frdA	4.13	0.0069	b4154	fumarate reductase (anaerobic) catalytic and NAD/flavoprotein subunit
aspA	4.12	0.0098	b4139	aspartate ammonia-lyase
dctR	4.10	0.0058	b3507	predicted DNA-binding transcriptional regulator
gatY	3.93	0.0124	b2096	D-tagatose 1,6-bisphosphate aldolase 2, catalytic subunit
wrbA	3.90	0.0125	b1004	NAD(P)H:quinone oxidoreductase flavoprotein WrbA (Trp repressor binding protein)
yneN	3.85	0.0040	b1500	Two component system connector membrane protein, EvgSA to PhoQP
pepT	3.84	0.0058	b1127	putative peptidase T
yjdK	3.84	0.0302	b4128	predicted protein
hypB	3.78	0.0129	b2727	GTP hydrolase involved in nickel liganding into hydrogenases
hypC	3.69	0.0138	b2728	protein required for maturation of hydrogenases 1 and 3
nikA	3.67	0.0325	b3476	nickel transporter subunit -!- periplasmic-binding component of ABC superfamily
dmsA	3.67	0.0144	b0894	anaerobic dimethyl sulfoxide reductase subunit A
emrY	3.64	0.0119	b2367	predicted multidrug efflux system
frdC	3.64	0.0077	b4152	fumarate reductase (anaerobic), membrane anchor subunit
udp	3.61	0.0183	b3831	uridine phosphorylase
yfdE	3.57	0.0058	b2371	predicted CoA-transferase, NAD(P)-binding
ogrK	3.57	0.0069	b2082	DNA-binding transcriptional regulator prophage P2 remnant
nrfC	3.56	0.0354	b4072	formate-dependent nitrite reductase, 4Fe4S subunit
hyaD	3.55	0.0255	b0975	processing of HyaA and HyaB proteins
dmsB	3.53	0.0269	b0895	anaerobic dimethyl sulfoxide reductase subunit B
yhbV	3.51	0.0082	b3159	predicted protease
yccJ	3.49	0.0108	b1003	predicted protein
srlA_1	3.44	0.0120	b2702	glucitol/sorbitol-specific enzyme IIC component of PTS
glpT	3.44	0.0103	b2240	sn-glycerol-3-phosphate transporter
srlE	3.43	0.0132	b2703	glucitol/sorbitol-specific enzyme IIB component of PTS
hyaB	3.43	0.0080	b0973	hydrogenase-1 large subunit
frdD	3.37	0.0127	b4151	fumarate reductase (anaerobic), membrane anchor subunit
malE	3.36	0.0197	b4034	maltose transporter subunit -!- periplasmic-

				binding component of ABC superfamily
napA	3.28	0.0233	b2206	nitrate reductase, periplasmic, large subunit
ysaA	3.28	0.0152	b3573	predicted hydrogenase, 4Fe-4S ferredoxin-type component
glpQ	3.27	0.0161	b2239	periplasmic glycerophosphodiester phosphodiesterase
ravA	3.25	0.0056	b3746	fused predicted transcriptional regulator: sigma54 activator protein/conserved protein putative 2-component regulator
gldA	3.25	0.0099	b3945	glycerol dehydrogenase, NAD
uspF	3.22	0.0132	b1376	putative filament protein
yjiY	3.21	0.0213	b4354	predicted inner membrane protein
nikC	3.19	0.0225	b3478	nickel transporter subunit transport of nickel, membrane protein; membrane component of ABC superfamily
manY	3.14	0.0104	b1818	mannose-specific enzyme IIC component of PTS
pepE	3.14	0.0266	b4021	(alpha)-aspartyl dipeptidase
adiY	3.14	0.0089	b4116	DNA-binding transcriptional activator
hyaC	3.12	0.0135	b0974	probable Ni/Fe-hydrogenase 1 b-type cytochrome subunit
hypD	3.11	0.0222	b2729	protein required for maturation of hydrogenases
ynfE	3.11	0.0087	b1587	oxidoreductase subunit
aegA	3.10	0.0089	b2468	fused predicted oxidoreductase: FeS binding subunit -!- NAD/FAD-binding subunit
yiiS	3.08	0.0026	b3922	conserved protein
manX	3.06	0.0088	b1817	fused mannose-specific PTS enzymes: IIA component -!- IIB component
uspE	3.03	0.0028	b1333	stress-induced protein
yghZ	2.98	0.0188	b3001	aldo-keto reductase
nikD	2.98	0.0457	b3479	nickel transporter subunit ATP-binding protein of nickel transport system; ATP-binding component of ABC superfamily
uspD	2.98	0.0054	b3923	stress-induced protein putative regulator
yieN	2.97	0.0027	b3746	fused predicted transcriptional regulator: sigma54 activator protein -!- conserved protein
bssR	2.97	0.0122	b0836	biofilm formation regulatory protein
nikB	2.96	0.0142	b3477	nickel transporter subunit transport of nickel, membrane protein; membrane component of ABC superfamily
manZ	2.96	0.0121	b1819	mannose-specific enzyme IID component of PTS
malP	2.95	0.0259	b3417	maltodextrin phosphorylase
ttdR	2.95	0.0386	b3060	putative transcriptional regulator LYSR-type
uspA	2.90	0.0040	b3495	universal stress global response regulator
yfbS	2.90	0.0027	b2292	predicted transporter
viaA	2.89	0.0013	b3745	predicted von Willibrand factor containing protein
ychH	2.86	0.0235	b1205	predicted inner membrane protein
cdd	2.78	0.0112	b2143	cytidine/deoxycytidine deaminase
ypdI	2.75	0.0134	b2376	predicted lipoprotein involved in colanic acid biosynthesis
ybaT	2.73	0.0141	b0486	putative amino acid/amine transport protein
ybaS	2.72	0.0096	b0485	putative glutaminase
yhbT	2.70	0.0088	b3157	predicted lipid carrier protein
yqgA	2.69	0.0203	b2966	predicted inner membrane protein

napG	2.66	0.0474	b2205	ferredoxin-type protein essential for electron transfer from ubiquinol to periplasmic nitrate reductase (NapAB)
dhaL	2.66	0.0031	b1199	dihydroxyacetone kinase
yjdO	2.66	0.0193	b4559	Predicted protein
ygcO	2.64	0.0044	b2767	predicted 4Fe-4S cluster-containing protein
yidF	2.64	0.0324	b3674	predicted DNA-binding transcriptional regulator
aphA	2.62	0.0193	b4055	acid phosphatase/phosphotransferase, class B, non-specific
yhdH	2.56	0.0134	b3253	predicted oxidoreductase, Zn-dependent and NAD(P)-binding
gutM	2.54	0.0242	b2706	DNA-binding transcriptional activator of glucitol operon
adhE	2.54	0.0290	b1241	CoA-linked acetaldehyde dehydrogenase/iron-dependent alcohol dehydrogenase
yeeR	2.54	0.0026	b2001	CP4-44 prophage; predicted membrane protein
deoA	2.53	0.0219	b4382	thymidine phosphorylase
yhbS	2.52	0.0030	b3156	predicted acyltransferase with acyl-CoA N-acyltransferase domain
dhaK	2.52	0.0026	b1200	dihydroxyacetone kinase
yaaJ	2.51	0.0127	b0007	putative inner membrane transport protein
ygaM	2.50	0.0155	b2672	predicted protein
hybO	2.48	0.0183	b2997	hydrogenase 2, small subunit
ynjE	2.44	0.0299	b1757	predicted thiosulfate sulfur transferase
rihA	2.43	0.0151	b0651	putative tRNA synthetase
treB	2.43	0.0289	b4240	fused trehalose(maltose)-specific PTS enzyme: IIB component -!- IIC component
zraS	2.41	0.0109	b4003	sensory histidine kinase in two-component regulatory system with ZraR
ykgE	2.39	0.0262	b0306	putative dehydrogenase subunit
dhaM	2.37	0.0136	b1198	fused predicted dihydroxyacetone-specific PTS enzymes: HPr component/EI component PTS hybrid protein
malT	2.37	0.0146	b3418	fused conserved protein -!- DNA-binding transcriptional activator, maltotriose-ATP-binding
hybA	2.36	0.0373	b2996	hydrogenase 2 4Fe-4S ferredoxin-type component
yccM	2.35	0.0134	b0992	predicted 4Fe-4S membrane protein
ygjR	2.33	0.0094	b3087	predicted NAD(P)-binding dehydrogenase
ygdH	2.32	0.0054	b2795	conserved protein
agp	2.31	0.0259	b1002	periplasmic glucose-1-phosphatase
yeaR	2.30	0.0228	b1797	conserved protein
yjjM	2.30	0.0106	b4357	predicted DNA-binding transcriptional regulator
yniA	2.30	0.0175	b1725	predicted phosphotransferase/kinase
ucpA	2.30	0.0102	b2426	predicted oxidoredutase, sulfate metabolism protein
deoC	2.29	0.0243	b4381	2-deoxyribose-5-phosphate aldolase, NAD(P)-linked
ybhG	2.27	0.0078	b0795	putative membrane protein
gadX	2.27	0.0028	b3516	DNA-binding transcriptional dual regulator
csiE	2.27	0.0186	b2535	stationary phase inducible protein
glk	2.26	0.0163	b2388	glucokinase
glpK	2.26	0.0025	b3926	glycerol kinase
ykgF	2.26	0.0334	b0307	predicted amino acid dehydrogenase with NAD(P)-binding domain and ferridoxin-like

				domain
crcA	2.25	0.0113	b0622	palmitoyl transferase for Lipid A
katG	2.24	0.0312	b3942	catalase/hydroperoxidase HPI(I)
yjiX	2.24	0.0169	b4353	conserved protein
copA	2.24	0.0083	b0484	Cu(I)-translocation P-type ATPase
yihM	2.24	0.0137	b3873	predicted sugar phosphate isomerase
yjjU	2.23	0.0021	b4377	predicted esterase
pfkA	2.23	0.0083	b3916	6-phosphofructokinase I
glgS	2.22	0.0127	b3049	predicted glycogen synthesis protein
nupG	2.20	0.0312	b2964	nucleoside transporter
rbsB	2.19	0.0103	b3751	D-ribose transporter subunit -!- periplasmic-binding compoent of ABC superfamily
rbsC	2.18	0.0026	b3750	D-ribose transporter subunit -!- membrane component of ABC superfamily
melR	2.17	0.0222	b4118	DNA-binding transcriptional dual regulator
treC	2.16	0.0249	b4239	trehalose-6-P hydrolase
yjiD	2.16	0.0060	b4326	DNA replication/recombination/repair protein
yibT	2.16	0.0063	b4554	Predicted protein
galP	2.15	0.0180	b2943	D-galactose transporter
ygcP	2.15	0.0085	b2768	predicted anti-terminator regulatory protein
yecH	2.15	0.0354	b1906	predicted protein
yjiM	2.14	0.0252	b4335	predicted 2-hydroxyglutaryl-CoA dehydratase
hcp	2.14	0.0456	b0873	putative prismane HCP protein
srlB	2.13	0.0327	b2704	glucitol/sorbitol-specific enzyme IIA component of PTS
yhiD	2.11	0.0030	b3508	predicted Mg(2+) transport ATPase inner membrane protein
yieE	2.10	0.0079	b3712	predicted phosphopantetheinyl transferase
yhiM	2.09	0.0077	b3491	conserved inner membrane protein
yahN	2.09	0.0403	b0328	putative cytochrome subunit of dehydrogenase
hyaF	2.08	0.0163	b0977	hydrogenase-1 cofactor biosynthesis protein hyaF
feoB	2.07	0.0228	b3409	fused ferrous iron transporter, protein B: GTP-binding protein -!- membrane protein
yagE	2.06	0.0077	b0268	CP4-6 prophage; predicted lyase/synthase
yjbQ	2.05	0.0004	b4056	conserved protein
ccmA	2.05	0.0383	b2201	heme exporter subunit -!- ATP-binding component of ABC superfamily
clpA	2.05	0.0146	b0882	ATP-binding component of serine protease
yjjB	2.03	0.0132	b4363	conserved inner membrane protein
gcvT	2.03	0.0034	b2905	aminomethyltransferase, tetrahydrofolate-dependent, subunit (T protein) of glycine cleavage complex
murQ	2.01	0.0279	b2428	N-acetylmuramic acid 6-phosphate etherase
uxaB	2.01	0.0163	b1521	altronate oxidoreductase, NAD-dependent
ygeV	2.01	0.0491	b2869	predicted DNA-binding transcriptional regulator
ybiH	2.00	0.0186	b0796	putative transcriptional regulator
dcuA	2.00	0.0131	b4138	C4-dicarboxylate antiporter
